# An Improved Red-Billed Blue Magpie Algorithm and Its Application to Constrained Optimization Problems

**DOI:** 10.3390/biomimetics10110788

**Published:** 2025-11-20

**Authors:** Ying Qiao, Zhixin Han, Hongxin Fu, Yuelin Gao

**Affiliations:** Ningxia Collaborative Innovation Center for Scientific Computing and Intelligent Information Processing, School of Mathematics and Information Science, North Minzu University, Yinchuan 750021, China; 15620696128@163.com (Z.H.); 15530163089@163.com (H.F.); 1993001@nun.edu.cn (Y.G.)

**Keywords:** metaheuristic, red-billed blue magpie algorithm, constrained optimization, red-billed blue magpie algorithm

## Abstract

The Red-Billed Blue Magpie Optimization (RBMO) algorithm is a metaheuristic method inspired by the foraging behavior of red-billed blue magpies. However, the conventional RBMO often suffers from premature convergence and performance degradation when solving high-dimensional constrained optimization problems due to its over-reliance on population mean vectors. To address these limitations, this study proposes an Improved Red-Billed Blue Magpie Optimization (IRBMO) algorithm through a multi-strategy fusion framework. IRBMO enhances population diversity through Logistic-Tent chaotic mapping, coordinates global and local search capabilities via a dynamic balance factor, and integrates a dual-mode perturbation mechanism that synergizes Jacobi curve strategies with Lévy flight strategies to balance exploration and exploitation. To validate IRBMO’s efficacy, comprehensive comparisons with 16 algorithms were conducted on the CEC-2017 (30D, 50D, 100D) and CEC-2022 (10D, 20D) benchmark suites. Subsequently, IRBMO was rigorously evaluated against ten additional competing algorithms across four constrained engineering design problems to validate its practical effectiveness and robustness in real-world optimization scenarios. Finally, IRBMO was applied to 3D UAV path planning, successfully avoiding hazardous zones while outperforming 15 alternative algorithms. Experimental results confirm that IRBMO exhibits statistically significant improvements in robustness, convergence accuracy, and speed compared to classical RBMO and other peers, offering an efficient solution for complex optimization challenges.

## 1. Introduction

For decades, optimization challenges have constituted a major research focus across diverse real-world domains. These problems center on maximizing or minimizing target functions while adhering to specific constraints. A model for a single-objective optimization problem can be represented by Equation (1).

(1) $$\begin{array}{cc} \; & Minimize:\;f\left(\vec{x}\right),\;\vec{x}=\left(x_{1},x_{2},..,x_{D}\right)\\ \; & Subject\;to:\\ \; & g_{i}\left(\vec{x}\right)\leq 0,\;i=1,...,N_{g}\\ \; & h_{j}\left(\vec{x}\right)=0,\;j=N_{g}+1,...,N_{g}+N_{h}\\ \; & lb_{u}\leq x_{u}\leq ub_{u},\;u\in 1,2,3,\ldots ,D \end{array}$$

In this context, $f(\vec{x})$ represents the objective function, where $\vec{x}$ is a *D*-dimensional vector consisting of the variables $x_{1}$ through $x_{D}$. The functions $g_{i}\left(\vec{x}\right)$ and $h_{j}\left(\vec{x}\right)$ represent the $N_{g}$ inequality constraints and $N_{h}$ equality constraints, respectively. The variables $lb_{u}$ and $ub_{u}$ denote the lower and upper bounds of $x_{u}$, respectively.

Optimization problems are ubiquitous across various fields, including industry, economics, and engineering design. However, with advancements in technology and human progress, existing methods are insufficient to address the increasingly complex optimization challenges. Therefore, identifying efficient and robust approaches for these complex optimization problems remains a significant challenge.

Optimization techniques are typically divided into two main types: deterministic methods and stochastic methods. Deterministic methods are typically based on mathematical models and seek optimal solutions to optimization problems through analytical approaches, offering strong theoretical foundations and precision. However, deterministic approaches often falter when applied to challenges characterized by complexity, multi-modality, and nonlinearity, particularly those that resist straightforward modeling. These methods are frequently plagued by significant issues, including high computational expenses and a tendency to become trapped in local optima. As a result, the efficacy of traditional optimization methods is increasingly constrained when confronted with the scale and complexity of modern problems. To overcome these challenges, stochastic optimization methods, particularly metaheuristic algorithms, have become a prominent focus of optimization research. Drawing inspiration from natural phenomena—such as biological, physical, and social behaviors—these approaches are characterized by robust global search capabilities and effective local optimization abilities. This synergy provides a significant advantage for addressing complex optimization challenges. While the vast majority of metaheuristic algorithms are designed for unconstrained optimization, they can also be applied to constrained problems by transforming them into an unconstrained format. Metaheuristic algorithms proposed over the past few decades are mainly classified into four categories: physics-based algorithms, evolution-based algorithms, human behavior-based algorithms, and swarm intelligence algorithms.

- **Physics-based algorithms**: These algorithms are inspired by natural phenomena, laws, and mechanisms in physics, leveraging phenomena such as motion, gravity, repulsion, and energy conservation between objects to develop optimization algorithms. For instance, Simulated Annealing (SA) [1], Circle Search Algorithm (CSA) [2], Geyser inspired Algorithm (GEA) [3], Big Bang-Big Crunch Algorithm (BBBC) [4], Subtraction-Average-Based Optimizer (SABO) [5], Elastic Deformation Optimization Algorithm (EDOA) [6], Kepler Optimization Algorithm (KOA) [7], Rime Optimizer (RIME) [8], Central Force Optimization (CFO) [9], Quadratic Interpolation Optimization (QIO) [10], Water Cycle Algorithm (WCA) [11], Sine Cosine Algorithm (SCA) [12], Homonuclear Molecules Optimization (HMO) [13], Turbulent Flow of Water Optimization (TFWO) [14], Newton’Raphson-Based Optimizer (NRBO) [15], Gradient-Based Optimizer (GBO) [16], Intelligent Water Drops Algorithm (IW-DA) [17], Equilibrium Optimizer (EO) [18], Fick’s Law Algorithm (FLA) [19], The Great Wall Construction Algorithm (GWCA) [20].
- **Evolution-based algorithms**: Inspired by Darwin’s theory of natural selection and genetic evolution, these algorithms model processes such as species evolution, survival of the fittest, genetic inheritance, and mutation, which collectively enhance the quality of a population over time. For example, Forest Optimization Algorithm (FOA) [21], Tree-Seed Algorithm (TSA) [22], Biogeography-Based Optimization (BBO) [23], Artificial Infectious Disease (AID) [24], Genetic Algorithm (GA) [25], Genetic Programming (GP) [26], Fungal Growth Optimizer (FGO) [27], Evolutionary Programming (EP) [28], Differential Evolution (DE) [29], Black Widow Optimization (BWO) [30], Human Felicity Algorithm (HFA) [31], Fungi Kingdom Expansion (FKE) [32].
- **Human behavior-based algorithms**: Inspired by human society, decision-making processes, learning, and psychological behavior patterns, these algorithms aim to find global optima by simulating human intelligence in decision-making, learning, collaboration, competition, and environmental adaptation. Typical human behavior-based algorithms include City Councils Evolution (CCE) [33], War Strategy Optimization (WSO) [34], Hiking Optimization Algorithm (HOA) [35], Teaching–Learning-Based Optimization (TLBO) [36], Political Optimizer (PO) [37], Volleyball Premier League (VPL) [38], Social Evolution and Learning Optimization (SELO) [39], Dream Optimization Algorithm (DOA) [40], Tabu Search (TS) [41], Football Team Training Algorithm (FTTA) [42], Soccer League Competition (SLC) [43], Brain Storm Optimization (BSO) [44], Kids Learning Optimizer (KLO) [45], Hunter Prey Optimization (HPO) [46], Exchange Market Algorithm (EMA) [47], Hunger Games Search (HGS) [48], Social Learning Optimization (SLO) [49], Mountaineering Team-Based Optimization (MTBO) [50],
- **Swarm Intelligence Algorithms**: These algorithms are inspired by the cooperative behavior of biological groups in nature. They achieve optimization tasks through simple interactions and collaboration between individuals. Notable swarm intelligence algorithms include Particle Swarm Optimization (PSO) [51], Zebra Optimization Algorithm (ZOA) [52], Moth-Flame Optimization (MFO) [53], Harris Hawks Optimization (HHO) [54], Artificial Rabbits Optimization (ARO) [55], Golden Jackal Optimization (GJO) [56], Ant Colony Algorithm (ACA) [57], Cuckoo Search (CS) [58], Snake Optimizer (SO) [59], Marine Predators Algorithm (MPA) [60], Secretary Bird Optimization algorithm (SBOA) [61], Artificial Gorilla Troops Optimizer (GTO) [62], Bat-inspired Algorithm (BA) [63], Whale Optimization Algorithm (WOA) [64], Gray Wolf Optimizer (GWO) [65], Sand Cat Swarm Optimization (SCSO) [66], Nutcracker Optimizer (NOA) [67], Aquila Optimizer (AO) [68], Black-winged Kite Algorithm (BKA) [69], Crested Porcupine Optimizer (CPO) [70], Hippopotamus Optimization Algorithm (HO) [71], African Vultures Optimization Algorithm (AVOA) [72], Sparrow Search Algorithm (SSA) [73], Dung Beetle Optimizer (DBO) [74], Honey Badger Algorithm (HBA) [75].

Using this established groundwork, a significant number of scholars have directed their attention toward improving the efficacy of contemporary algorithms. Among these, PSO [51] has gained significant attention as one of the most widely studied swarm intelligence optimization algorithms in recent years, owing to its efficiency. Although traditional PSO exhibits robust global optimization capabilities, it is prone to premature convergence. To address this issue, many researchers have proposed modifications to PSO, resulting in the development of various improved or even significantly enhanced variants. As an example, Zhi et al. [76] proposed the Adaptive Multi-Population Particle Swarm Optimization (AMP-PSO) algorithm as a solution for the cooperative path planning challenge involving several Autonomous Underwater Vehicles (AUVs). This algorithm incorporated a multi-population grouping strategy and an interaction mechanism among the particle swarms. By dividing particles based on their fitness, one leader swarm and several follower swarms were established, significantly improving the optimization performance. Huang et al. [77] developed the ACVDEPSO algorithm, in which particle velocity was represented as cylindrical vectors to facilitate path searching. Moreover, this work incorporates a challenger mechanism that leverages differential evolution operators. The purpose of this addition is to diminish the algorithm’s susceptibility to local optima, which consequently yields a substantial acceleration in convergence. Li et al. [78] proposed the Pyramid Particle Swarm Optimization (PPSO) algorithm, which employed a pyramid structure to allocate particles to different levels according to their fitness. Within each level, particles competed in pairs to determine winners and losers. Losers collaborated with their corresponding winners to optimize performance, while winners interacted with particles from higher levels, including the top level of the pyramid. This methodology bolstered the algorithm’s capacity for global exploration.

Similar to PSO, GWO [65] has also emerged as a widely applied swarm intelligence optimization algorithm in recent years. GWO derives its core concepts from the communal structure and predatory patterns of grey wolves. This approach has achieved widespread adoption as a technique for addressing optimization challenges across numerous engineering disciplines. For instance, Qiu et al. [79] introduced an Improved Grey Wolf Optimizer (IGWO) that integrates multiple strategies to enhance performance. The algorithm employs a Lens Imaging-based Opposition Learning method and a nonlinear convergence strategy, using cosine variation to control parameters, thus achieving a balance between global exploration and local exploitation. Additionally, drawing inspiration from the Tentacle-inspired Swarm Algorithm (TSA) [80] and PSO, a nonlinear parameter adjustment strategy was incorporated into the position update equation. This was further combined with corrections based on both individual historical best positions and global best positions, which improved convergence efficiency. Zhang et al. [81] proposed an enhanced version of GWO, known as VGWO-AWLO, which incorporates velocity guidance, adaptive weights, and the Laplace operator. The algorithm first introduces a dynamic adaptive weighting mechanism based on uniform distribution, enabling the control parameter *a* to achieve nonlinear dynamic variation, facilitating smooth transitions between exploration and exploitation phases. Second, a novel velocity-based position update equation was developed, along with an individual memory function to enhance local search capabilities and accelerate convergence toward the optimal solution. Third, a Laplace crossover operator strategy was employed to further enhance population diversity, effectively preventing the grey wolf population from becoming trapped in local optima. MELGWO, an enhanced variant of GWO, was introduced by Ahmed et al. [82]. The novel method incorporates a hybrid of memory mechanisms, evolutionary operators, and stochastic local search techniques. Additionally, the algorithm incorporates Linear Population Size Reduction (LPSR) [83] technology to further boost performance. Beyond PSO and GWO, many researchers have also focused on improving other swarm intelligence optimization algorithms to enhance their efficiency and effectiveness, though these advancements are not discussed in detail here.

Among the numerous metaheuristic algorithms, Differential Evolution (DE) [29] stands out. Proposed by Storn and Price in the mid-1990s, DE utilizes difference vectors between individuals for mutation, integrating this with crossover and selection to guide the population search. DE is renowned for its simple structure, strong robustness, and superior convergence performance. Its powerful performance has laid a foundation for the subsequent development of metaheuristic algorithms.

In the decades that followed, numerous DE variants continued to demonstrate superior performance. In 2009, SaDE (Self-Adaptive Differential Evolution) [84], an improved variant of DE, won the CEC competition. The key feature of SaDE is its ability to self-adapt its control parameters, primarily the scaling factor *F* and crossover probability CR. The scaling factor *F* governs the magnitude of the mutation, while the crossover probability CR controls the degree of recombination. This adaptive mechanism allows SaDE to tailor its behavior to different problems without manual parameter tuning, thereby reducing the cost of human intervention.

In 2014, LSHADE [85], another DE variant, won the CEC competition. The LSHADE algorithm marked a significant milestone in the evolution of DE. LSHADE introduced a linear population reduction mechanism, which further enhanced convergence performance and solidified the dominance of DE-based algorithms in subsequent competitions.

In 2017, LSHADE-cnEpSin [86] emerged as one of the winners of the CEC-2017 competition. As an enhancement of LSHADE, this algorithm employs a parameter adaptation strategy adjusted by a sinusoidal function, enabling it to exhibit different behaviors at various search stages. A more effective equilibrium between the algorithm’s global exploration and local exploitation phases is achieved through this framework. Within the LSHADE framework, more high-performing variants were subsequently proposed. For example, LSHADE_SPACMA [87] is another notable advanced variant, frequently featured as a state-of-the-art representative in recent benchmark comparisons. First, the algorithm employs a dedicated elimination and generation mechanism specifically to bolster its local search intensification. Additionally, the algorithm leverages a mutation operator built upon an enhanced semi-parametric adaptation method and rank-based selection pressure. This combination is designed to effectively steer the evolutionary path. Moreover, LSHADE_SPACMA features an elite-based external archive, a component tasked with both preserving external solution diversity and promoting faster convergence.

The strong momentum of the DE algorithm family did not wane. IUDE [88], jDE100 [89], and IMODE [90] subsequently won the CEC competitions in 2018, 2019, and 2020, respectively. The continuous evolution of these CEC-winning algorithms clearly demonstrates the robustness and longevity of the DE framework, as well as its exceptional potential for solving complex real-number optimization problems.

The Red-billed Blue Magpie Optimizer (RBMO) is a novel swarm intelligence optimization algorithm introduced by Xun et al. in 2024 [91]. The algorithm was successfully applied to the 3D path planning problem for UAVs, yielding impressive results. To achieve its optimization goals, RBMO models the primary foraging strategies of the red-billed blue magpie, namely its techniques for finding food, attacking prey, and caching supplies. However, the algorithm’s proven effectiveness is constrained by an insufficient global search capability, which creates a vulnerability to premature convergence in local optima. To address these shortcomings, Zhang et al. [92] introduced an improved version of the algorithm, known as ES-RBMO, which incorporates an elite strategy. This enhanced version aims to mitigate the issue of local optima by preserving low-fitness individuals through the elite strategy. However, although Zhang et al. emphasized that ES-RBMO effectively reduces the likelihood of becoming trapped in local optima, it does not resolve the inherent limitations in RBMO’s search expressions, which can still lead to difficulties in escaping local optima.

Despite the continuous emergence of new metaheuristic algorithms and improved versions of existing algorithms in recent years, according to the “No Free Lunch” theorem [93], no single algorithm is universally applicable to all problems. Therefore, it remains necessary to continue searching for a metaheuristic algorithm capable of solving the majority of optimization problems. To address the limitations of the classical RBMO algorithm, expand its application scope, and identify methods capable of solving a broader range of optimization problems, this paper proposes an improved version of the RBMO algorithm, termed IRBMO. First, IRBMO introduces a balance factor that dynamically adjusts the weight between the mean and the global optimum, enabling particles to effectively obtain optimal state information from the population, thereby accelerating convergence and improving solution adaptability. Second, it incorporates multiple strategies to enhance population diversity. Third, Jacobi curves [94] and Lévy Flight [95] are integrated into the search phase to mitigate RBMO’s inability to escape local optima, significantly improving its ability to avoid premature convergence.

Experimental results on the CEC benchmark set, real-world engineering constrained optimization problems, and 3D UAV path planning in mountainous terrain demonstrate that IRBMO significantly improves upon the classical RBMO, achieving satisfactory results. In this paper, we highlight the following key contributions:

- Improving Escape from Local Optima: To address the drawbacks of the classical RBMO algorithm, such as premature convergence, excessive reliance on the mean, and the inability to escape local optima, IRBMO is proposed.
- Comprehensive Benchmark Testing: The performance of 16 algorithms, including award-winning CEC competition algorithms, widely used algorithms or their improved versions, recently proposed high-performance and highly cited algorithms, as well as the classical RBMO and the proposed IRBMO, is compared on the CEC-2017 test sets of 30, 50, and 100 dimensions and the CEC-2022 test sets of 10 and 20 dimensions. To evaluate the performance of the 16 algorithms, radar charts, heat maps, Sankey diagrams, stacked bar charts, and line charts were employed to visualize their rankings and Friedman mean ranks across various test sets. We employed the Wilcoxon rank-sum test to ascertain if IRBMO’s performance was statistically distinct from the other algorithms. Additionally, we generated box plots to visualize the performance stability of each method. The findings from the CEC test sets reveal that IRBMO surpassed the other 15 algorithms on a majority of test functions, highlighting its superior stability, robustness, effectiveness, and capacity to avoid local optima.
- Real-World Constrained Engineering Problems: The performance of 11 algorithms was evaluated across four real-world engineering constrained problems to validate the potential of IRBMO in addressing such challenges.
- 3D Path Planning for UAVs: IRBMO is applied to the 3D path planning problem for UAVs, and its performance is compared against 15 algorithms. Results validate IRBMO’s competitive edge and suitability for tackling 3D path planning problems relative to other state-of-the-art algorithms.

The IRBMO algorithm effectively mitigates several inherent limitations of traditional path planning methods, including navigation challenges in complex terrain environments and susceptibility to local optima. Compared to existing approaches, IRBMO demonstrates notable advancements in UAV path planning, achieving higher computational efficiency and operational feasibility. These improvements address critical gaps in prior research while offering practical solutions to persistent challenges in path planning methodologies.

## 2. The Red-Billed Blue Magpie Optimizer

The Red-Billed Blue Magpie Optimizer (RBMO) is a novel swarm intelligence optimization algorithm proposed by Xun et al. in 2024 [91].

This algorithm emulates the foraging strategies of Red-Billed Blue Magpies—specifically, searching for food, attacking prey, and storing it—to achieve a global search for the optimal solution. The original authors demonstrated RBMO’s robust performance by evaluating it on the CEC-2014 [96] and CEC-2017 benchmark test sets, using dimensions of 10, 30, 50, and 100. The comparisons were made against high-performing champion algorithms such as LSHADE-cnEpSin [86] and EBOwithCMAR [97], as well as highly cited algorithms like COA and DBO.

### 2.1. Population Initialization

RBMO first establishes a population of *N* individuals located within the solution space. Each of these individuals is defined by a solution vector that contains multiple dimensions. For instance, in a *D*-dimensional optimization problem, the position of the $i^{th}$ Red-Billed Blue Magpie is expressed as $X_{i}=\left[x_{i,1},x_{i,2},\ldots ,x_{i,D}\right]$. The entire population, consisting of *N* Red-Billed Blue Magpies, can be represented as Equation (2).

(2) $$X={\left[\begin{array}{c} X_{1}\\ \vdots \\ X_{i}\\ \vdots \\ X_{N} \end{array}\right]}_{N\times 1}=\left[\begin{array}{ccc} x_{1,1} & \ldots & x_{1,D}\\ \vdots & \ddots & \vdots \\ x_{N,1} & \ldots & x_{N,D} \end{array}\right]$$

Here, *N* represents the population size, *D* denotes the problem’s dimensionality, and X represents the matrix formed by the population of size *N*. Each element $x_{i,j}$ in the matrix is a random number generated as part of Equation (3).

(3) $$x_{i,j}=\left(ub_{j}-lb_{j}\right)\times Rand_{1}+lb_{j}\;,\;i\in 1,2,3,\ldots ,N\;,\;j\in 1,2,3,\ldots ,D$$

Here, $ub_{j}$ and $lb_{j}$ denote the upper and lower bounds of the $j^{th}$ dimension in the solution space, respectively, and $Rand_{1}$ denotes a random number drawn from a uniform distribution over the interval [0,1]. Each agent in the Red-Billed Blue Magpie population represents a candidate solution to the optimization problem. The objective function of each solution vector is computed as follows, forming an N-dimensional fitness matrix *F*:

(4) $${\left.F=\left[\begin{array}{c} fit_{1}\\ \vdots \\ fit_{i}\\ \vdots \\ fit_{N} \end{array}\right.\right]}_{N\times 1}={\left[\begin{array}{c} f(X_{1})\\ \vdots \\ f(X_{i})\\ \vdots \\ f(X_{N}) \end{array}\right]}_{N\times 1}$$

where $fit_{i}$ denotes the fitness value of the $i^{th}$ agent. In minimization problems, solutions with smaller fitness values indicate superior quality, whereas the inverse holds for maximization problems. The global optimum $X^{food}$ is identified by selecting the solution vector with the highest fitness quality through systematic evaluation.

### 2.2. The Mathematical Model of RBMO

#### 2.2.1. Search for Food

To enhance foraging efficiency, red-billed blue magpies typically search for food by forming small groups (2 to 5 individuals) or even larger flocks (exceeding 10). A variety of techniques are employed by them, which involve walking and jumping on the ground, as well as exploring trees to locate food sources. This adaptability and flexibility enable the red-billed blue magpies to adopt diverse hunting methods adjusted to resource availability and environmental conditions, thus securing an adequate supply of food. Use Equation (5) when small groups are exploring food, and apply Equation (6) during cluster foraging.

(5) $$X_{i}\left(t+1\right)=X_{i}\left(t\right)+\left(\frac{1}{p}\times \sum_{m=1}^{p}X^{m}\left(t\right)-X^{rs}\left(t\right)\right)\times Rand_{2}$$

(6) $$X_{i}\left(t+1\right)=X_{i}\left(t\right)+\left(\frac{1}{q}\times \sum_{m=1}^{q}X^{m}\left(t\right)-X^{rs}\left(t\right)\right)\times Rand_{3}$$

Here, *t* represents the current iteration count, $X_{i}\left(t+1\right)$ signifies the position of the $i^{th}$ new search agent, and $X_{i}$ denotes the position of the $i^{th}$ individual. $Rand_{2}$ and $Rand_{3}$ correspond to independent uniformly distributed random numbers within the interval [0,1]. The parameter *p* represents the quantity of red-billed blue magpies foraging within a small group. This value is chosen at random from an integer range of 2 to 5. Meanwhile, *q* represents the number of red-billed blue magpies randomly chosen for cluster foraging, with a group size between 10 and *N*. $X^{m}$ marks the position of the $m^{th}$ individual within the randomly selected small group or cluster, and $X^{rs}\left(t\right)$ indicates a search agent randomly selected in the current iteration. The original text equally divides the probability of using Equations (5) and (6) through a random number to balance the frequency of employing small groups and clusters.

#### 2.2.2. Attacking Prey

Red-billed blue magpies display exceptional hunting prowess and teamwork during prey pursuit. Their methods involve swift pecking, pouncing to seize prey, or snagging insects mid-air. When foraging in small teams, their objectives are usually minor prey or flora; this behavior is mathematically modeled in Equation (7). Conversely, operations in larger flocks allow for cooperative assaults on more substantial targets, like large invertebrates or small mammals, which is detailed in Equation (8). This hunting conduct underscores their strategic diversity and proficiency, establishing them as adaptable predators capable of feeding in myriad contexts.

(7) $$X_{i}\left(t+1\right)=X^{food}\left(t\right)+CF\times \left(\frac{1}{p}\times \sum_{m=1}^{p}X^{m}\left(t\right)-X_{i}\left(t\right)\right)\times Rand_{n1}$$

(8) $$X_{i}\left(t+1\right)=X^{food}\left(t\right)+CF\times \left(\frac{1}{q}\times \sum_{m=1}^{q}X^{m}\left(t\right)-X_{i}\left(t\right)\right)\times Rand_{n2}$$

$$CF={\left(\begin{array}{c} 1-\left(\begin{array}{c} \frac{t}{T} \end{array}\right) \end{array}\right)}^{\left(2\times \frac{t}{T}\right)}$$

where $X^{food}\left(t\right)$ denotes the position of the food source, which represents the best solution found so far during the search process. *T* represents the maximum number of iterations, and Randn denotes a random number generated from a standard normal distribution. Similarly, in this stage, the probabilities of using Equations (7) and (8) are also equally divided.

#### 2.2.3. Food Storage

Beyond just seeking and capturing sustenance, red-billed blue magpies also hoard surplus provisions in tree hollows or other hidden spots for future consumption. This caching behavior ensures a stable source of nourishment during times of scarcity. The approach also retains solution information, helping individuals identify the global optimum. The associated mathematical model is presented in Equation (9).

(9) $$X_{i}\left(t+1\right)=\left\{\begin{array}{ccc} X_{i}\left(t\right) & \; & if\;fit_{i}^{old}>fit_{i}^{new}\\ X_{i}\left(t+1\right) & \; & else \end{array}\right.$$

Here, $fit_{i}^{old}$ and $fit_{i}^{new}$ denote the fitness values for the $i^{th}$ red-billed blue magpie prior to and following its location update, respectively.

**Figure 1:**
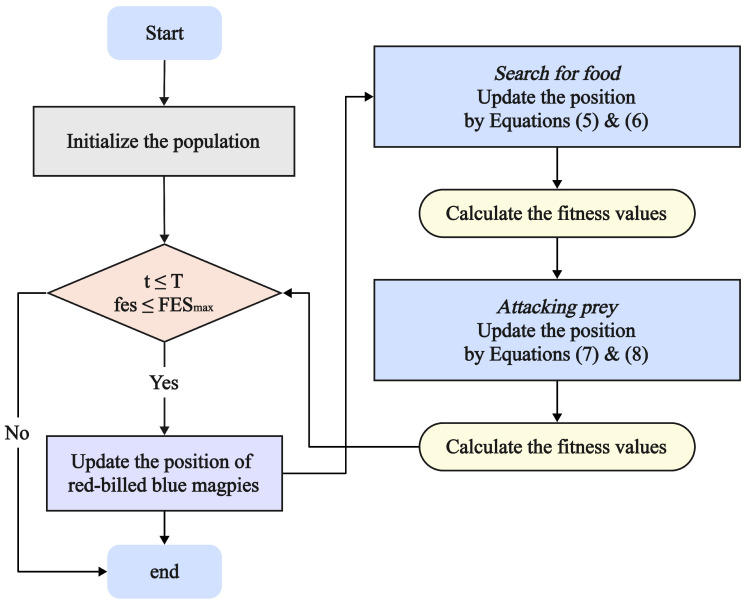
The flowchart of RBMO algorithm.

The RBMO flowchart and pseudocode are presented in Figure 1 and Algorithm 1.
**Algorithm 1** Pseudocode of RBMO.  1:**Input:** Population size *N*, Dimension *D*, lower bounds lb and upper bounds ub, Maximum number of Iterations *T* and Maximum number of Evaluation $FES_{max}$.  2:**Output:** The minimum fitness $fit^{food}$ and the best solution $X^{food}$  3:**Initialization:** Initial population X and fitness F by Equations (2)–(4).  4:**while**  $t\;\leq \;T\;\&\;fes\;\leq \;FES_{max}$  **do**  5:   Update population X by Equations (5) and (6)  6:   Calculate fitness F and update $fit^{food}$ and $X^{food}$ by Equation (9)  7:   Update population X by Equations (7) and (8)  8:   Calculate fitness F and update $fit^{food}$ and $X^{food}$ by Equation (9)  9:   t=t+1&fes=fes+2N10:**end while**11:**Return** The minimum fitness $fit^{food}$ and the best solution $X^{food}$

## 3. Improving the Red-Billed Blue Magpie Optimizer

### 3.1. Reasons for Improvements

Although the original text has demonstrated the robust performance of RBMO, further research has revealed certain limitations. To fully understand these limitations, it is essential to first conduct a critical conceptual comparison of RBMO with other prominent metaheuristics such as PSO, GWO, DE, and AVOA. This analysis will not only highlight RBMO’s unique search mechanism but also expose the inherent vulnerabilities that motivate our proposed improvements.

RBMO’s novelty stems from a search mechanism that is conceptually distinct from these established algorithms. However, these foundational differences are also the source of its specific weaknesses. The conceptual comparison is as follows:

- **PSO:** It updates particles based on two critical memory components: the particle’s own best-known position ($p_{best}$) and the swarm’s global best position ($g_{best}$). Conceptually, RBMO lacks the $p_{best}$ component. Its search phase (Equations (5) and (6)) relies on a stochastic subgroup mean as social information, rather than a particle’s individual experience.
- **GWO:** It is guided by a social hierarchy composed of the three best solutions (α,β,δ). This leadership committee guides the search. RBMO, in contrast, only utilizes a single global best ($X^{food}$) during its attacking prey phase (Equations (7) and (8)) and relies on a peer-group mean, not leaders, during its search for food phase.
- **DE:** It creates mutant vectors using difference vectors between randomly sampled individuals. While RBMO also uses difference vectors, its core operator is conceptually different, as it computes the difference between a subgroup mean and another individual.
- **AVOA:** It features multiple, complex, and distinct behavioral phases (starvation, siege, revolving flight), each with different mathematical models. RBMO’s structure is simpler and more unified, with both its search and attack phases being variations built upon the same core concept: the subgroup mean.

This critical comparison reveals that RBMO’s primary conceptual characteristic is its heavy reliance on the mean of a random subgroup. This very mechanism, which defines RBMO, is also the source of its fundamental limitations.

First, like many metaheuristics, its performance is sensitive to the initial population. The uneven distribution of the randomly initialized population can reduce global search capability.

Second, and more critically, the aforementioned excessive reliance on the mean (a direct consequence of its core concept) makes it difficult to escape local optima. As the population converges and diversity is lost, the *mean* of any random subgroup becomes almost identical to any given individual ($X^{rs}\left(t\right)$ or $X_{i}\left(t\right)$). Consequently, the core difference vectors in Equations (5) through (8) mathematically converge to zero. This stagnation stops the search process, trapping the algorithm in a local optimum and causing premature convergence.

To address these specific limitations, this paper proposes the following three improvements.

### 3.2. Optimizing the Initial Population

The efficiency of RBMO is significantly influenced by the initialization of the population. The ability to significantly widen the exploration domain is a key benefit of an evenly dispersed population, which in turn bolsters the convergence rate and solution precision. Regrettably, conventional RBMO methods adopt a purely stochastic method for initializing the population. This technique frequently yields aggregated starting points, resulting in poor coverage and a non-uniform spread of individuals across the problem space. Consequently, this diminishes population diversity, adversely affecting the convergence speed and accuracy of the algorithm. Therefore, we consider utilizing chaotic mapping [98] to achieve a more evenly distributed initial population, aiming to enhance the algorithm’s performance.

Among the spectrum of chaotic maps, the tent map [99] is often selected for its straightforward formulation and its capacity to produce uniformly distributed sequences. It demonstrates robust ergodic properties and its design facilitates simple integration with other algorithmic frameworks. On the other hand, the Logistic chaotic map [100] is represented by a simple nonlinear equation, characterized by good discreteness, strong randomness, low computational cost, and ease of implementation, making it widely used across multiple fields.

**Figure 2:**
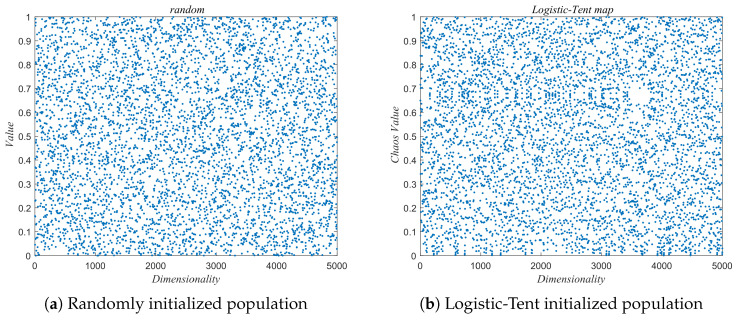
Comparative Analysis of Initialized Populations.

The Logistic-tent chaotic map combines the dynamic characteristics of both the Logistic and tent maps to create a novel system. While maintaining simplicity, this system exhibits more complex dynamical behavior. This map synthesizes the strong sensitivity of the Logistic map with the periodic features of the tent map. This combination yields sequences possessing enhanced randomness and broader diversity. For this study, the Logistic-tent chaotic map is utilized to generate the initial population, with its mathematical definition provided as follows:

(10) $$x_{i+1,u}=\left\{\begin{array}{c} mod\left(rx_{i,u}\left(1-x_{i,u}\right)+\left(4-r\right)x_{i,u}/2,1\right),\;x_{i}<0.5\\ mod\left(rx_{i,u}\left(1-x_{i,u}\right)+\left(4-r\right)\left(1-x_{i,u}\right)/2,1\right),\;x_{i}\geq 0.5 \end{array}\right.\;u\in 1,2,3,\ldots ,D$$

where *x* represents the variable and *r* is the control parameter, with *x* ranging between 0 and 1, and *r* spanning from 0 to 4. The improved IRBMO uses the logistic-tent chaotic map for population initialization, which ensures that the initial solutions are as uniformly distributed as possible within the solution space, as shown in Figure 2.

### 3.3. Introduction of a Balance Factor

To prevent excessive reliance on the mean, we introduce a balance factor λ and the position of the food source $X^{food}\left(t\right)$ from the classic RBMO (i.e., the known optimal solution). The weights assigned to the mean and the global optimum are adjusted dynamically in accordance with the current iteration’s progress. This strategy allows particles to more effectively assimilate the population’s optimal state information, which in turn promotes a faster convergence process. The improved expressions for the Search for food stage are shown in Equations (11), (12) and (14).

(11) $$X_{i}\left(t+1\right)=X_{i}\left(t\right)+\left[\lambda \times \left(\frac{1}{p}\times \sum_{m=1}^{p}X^{m}\left(t\right)-X^{rs}\left(t\right)\right)+\left(1-\lambda \right)\times \left(X^{food}\left(t\right)-X^{rs}\left(t\right)\right)\right]\times r$$

(12) $$X_{i}\left(t+1\right)=X_{i}\left(t\right)+\left[\lambda \times \left(\frac{1}{q}\times \sum_{m=1}^{q}X^{m}\left(t\right)-X^{rs}\left(t\right)\right)+\left(1-\lambda \right)\times \left(X^{food}\left(t\right)-X^{rs}\left(t\right)\right)\right]\times r$$

(13) $$\lambda =1-0.5\times {\left(t/T\right)}^{2}$$

(14) $$r=\left\{\begin{array}{cc} Rand_{2} & Rand_{3}<1-{\left(\frac{t}{T}\right)}^{4}\\ \sin(Rand_{2}) & Rand_{3}\geq 1-{\left(\frac{t}{T}\right)}^{4}\;and\;Rand_{4}>0.5\\ \cos(Rand_{2}) & else \end{array}\right.$$

In this context, *T* denotes the total number of iterations. Meanwhile, $Rand_{2}$, $Rand_{3}$ and $Rand_{4}$ are random numbers drawn independently from a uniform distribution over the interval [0,1]. In this context, the probabilities of adopting Equations (11) and (12) remain unchanged (consistent with the original Search for food stage).

### 3.4. Jacobi Curve and Lévy Flight

Although RBMO demonstrates strong performance, a critical analysis of its update expressions reveals an inherent vulnerability. As the population converges, the differential vectors that propel the search—namely $\left(\frac{1}{p}\times \sum_{m=1}^{p}X^{m}\left(t\right)-X^{rs}\left(t\right)\right)$ and others in Equations (5)–(8)—inevitably diminish towards zero. This mathematical property leads to the stagnation of the search process, trapping the algorithm in local optima and precluding further exploration. To fundamentally address this issue, we propose a sophisticated hybrid perturbation mechanism that strategically integrates the Jacobi Curve and Lévy Flight.

The core design philosophy is to establish a dual-mode perturbation strategy where two distinct forms of stochasticity complement each other. On one hand, we replace the standard random number at the end of the Attacking Prey phase equations with a Lévy Flight function LF, as detailed in Equations (15) and (16). This mechanism models a scale-free random walk, characterized by intermittent, long-range jumps resulting from its heavy-tailed probability distribution. This grants the algorithm a powerful broad-area reconnaissance capability, enabling it to launch exploratory probes into distant regions of the search space and discover novel solutions far from the current point of convergence.

This leads to the modified update rules:

(15) $$X_{i}\left(t+1\right)=X^{food}\left(t\right)+CF\times \left(\frac{1}{p}\times \sum_{m=1}^{p}X^{m}\left(t\right)-X_{i}\left(t\right)\right)\times LF$$

(16) $$X_{i}\left(t+1\right)=X^{food}\left(t\right)+CF\times \left(\frac{1}{q}\times \sum_{m=1}^{q}X^{m}\left(t\right)-X_{i}\left(t\right)\right)\times LF$$

On the other hand, the Jacobi Curve is introduced as a specialized mutation operator to generate a fundamentally different type of perturbation. As formulated by Li et al. [94] and adopted in Equation (17), this operator, while incorporating random variables ($Rand_{5}$ and θ), is not an unstructured random walk. Instead, it generates a structured stochastic perturbation, where the random elements guide a search along a specific nonlinear trajectory defined by the curve’s mathematical structure. This results in a more methodical, formula-guided escape attempt from the local neighborhood of the current best solution.

(17) $$X_{i}\left(t+1\right)=Rand_{5}\times X_{i}\left(t\right)+\left(e^{\theta }-2\times X^{food}\left(t\right)\times \sin\theta \right)/\left(\sin\theta -\cos\theta \right)$$

where $Rand_{5}$ follows a uniform distribution in the interval [0,1]; θ is a control parameter restricted to the domain [0,π].

The proposed mechanism operates probabilistically: if a randomly generated number $Rand_{6}<0.05$, Equation (17) is activated. Otherwise, the algorithm selects between Equations (15) and (16) with equal likelihood. This hybrid design harmonizes two distinct stochastic strategies: the unbounded, scale-free exploration of Lévy Flight and the model-guided, structured perturbations of the Jacobi Curve. By strategically combining these complementary search paradigms, the algorithm achieves enhanced robustness from local optima escape while maintaining stable convergence properties. Unlike PSO’s reliance on personal-best and global-best memory, or GWO’s hierarchical leadership, RBMO’s dependence on subgroup means necessitates a tailored diversification strategy. The Jacobi–Lévy hybrid, while inspired by similar mechanisms in AVOA and DBO, is uniquely designed to disrupt RBMO’s mean-driven stagnation. This strategic integration of global and local perturbations is absent in prior RBMO variants and distinguishes IRBMO from other metaheuristics.

**Figure 3:**
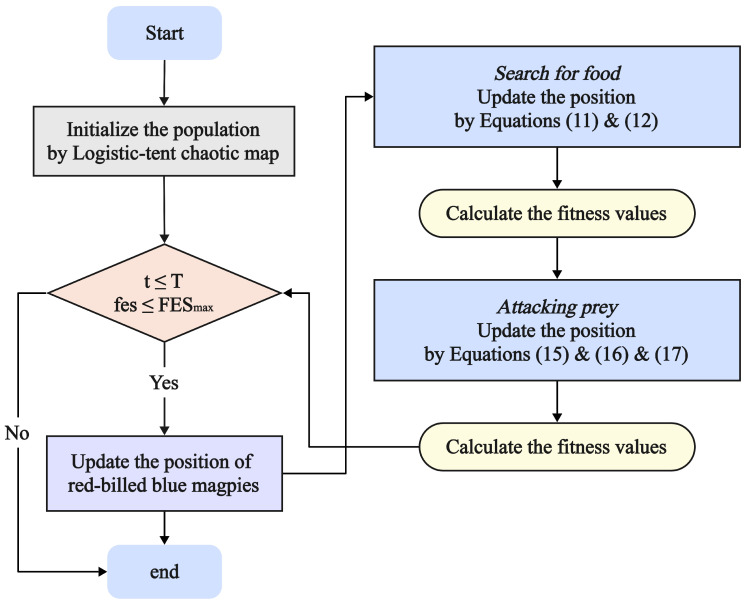
The flowchart of IRBMO algorithm.

The IRBMO flowchart and pseudocode are presented in Figure 3 and Algorithm 2.
**Algorithm 2** Pseudocode of IRBMO.  1:**Input:** Population size *N*, Dimension *D*, lower bounds lb and upper bounds ub, Maximum number of Iterations *T* and Maximum number of Evaluation $FES_{max}$.  2:**Output:** The minimum fitness $fit^{food}$ and the best solution $X^{food}$  3:**Initialization:** Initial population X and fitness F by Equations (10), (3) and (4).  4:**while**  $t\;\leq \;T\;\&\;fes\;\leq \;FES_{max}$  **do**  5:   Update population X by Equations (11) and (12)  6:   Calculate fitness F and update $fit^{food}$ and $X^{food}$ by Equation (9)  7:   Update population X by Equations (15)–(17)  8:   Calculate fitness F and update $fit^{food}$ and $X^{food}$ by Equation (9)  9:   t=t+1&fes=fes+2N10:**end while**11:**Return** The minimum fitness $fit^{food}$ and the best solution $X^{food}$

### 3.5. Time Complexity Analysis

In this section, we will conduct a comparative analysis of the time complexity between RBMO and IRBMO. The parameters are defined as follows: *N* indicates the total count of search agents, *T* specifies the maximum iteration limit, and *D* corresponds to the dimension of the problem under consideration. The original text analyzes the time complexity from two perspectives: solution initialization and solution update processes, concluding that the time complexity of RBMO is O(N×T×(2×D+1)). To ensure a fair comparison, this analysis also considers these two aspects.

Initially, during the solution initialization phase, the complexity of RBMO is O(N). The IRBMO introduces an additional complexity of O(N) due to the incorporation of the Logistic-tent chaotic map. Therefore, the overall complexity for IRBMO is O(2N), which is an increase of O(N) compared to RBMO.

Secondly, IRBMO evaluates the fitness function the same number of times as RBMO, and the frequency of updating the positions of search agents is not increased. Consequently, the time complexity for this part remains the same as that of RBMO, which is O(T×N)+O(2×T×N×D).

Throughout the entire process, IRBMO only introduces an additional complexity of O(N) during the solution initialization phase compared to RBMO. Thus, the time complexity of IRBMO is O(N×(T×(2×D+1)+1)). This indicates that while enhancing performance, IRBMO incurs only a minimal increase in complexity.

## 4. The Results of the IRBMO Algorithm on Different Test Sets

In this section, we validate the potential of IRBMO in solving optimization problems by thoroughly discussing its performance on the CEC benchmark test suites, as compared to other competitive algorithms. Section 4.1 presents the experimental setup, Section 4.2 validates the effectiveness of the proposed strategy and conducts a parameter sensitivity analysis, Section 4.3 analyzes the convergence behavior of IRBMO, Section 4.4 and Section 4.5 provide a detailed discussion of the experimental results for IRBMO and 15 other algorithms on the CEC-2017 and CEC-2022 test suites.

### 4.1. Experimental Design

#### 4.1.1. Competing Algorithms and Parameter Settings

This section presents a comparative performance evaluation of 16 algorithms, benchmarked against the CEC test suites. The methods selected for this study are as follows:

- The classic RBMO and the IRBMO proposed in this paper.
- The award-winning algorithms in the CEC competition, LSHADE-cnEpSin and LSHADE_SPACMA.
- Traditional and widely recognized metaheuristic algorithms or their improved versions: PPSO, MELGWO, and WOA.
- Recently proposed high-performance, highly cited metaheuristic algorithms include: SSA, GJO, AVOA, SO, DBO, GTO, BKA, HO, and RIME.

Table 1 contains the hyperparameters of the algorithms mentioned. The experimental evaluation was conducted using problems from two CEC benchmarks. Specifically, the CEC-2017 suite provided instances at 30, 50, and 100 dimensions, while the CEC-2022 suite supplied 10 and 20-dimensional problems. For parameter configuration, the population size was uniformly set to 30, with the termination criterion defined as 30,000 maximum fitness evaluations. To ensure statistical validity, 30 independent runs were executed for each algorithm. The complete statistical outcomes, encompassing the mean, standard deviation (Std), best, and worst values for every test suite, are detailed in Table S1. To visually present the comparison results, the last row of the table summarizes the number of wins, ties, and losses of IRBMO compared to the target algorithm based on the mean values. Here, “W” indicates the number of times IRBMO outperformed the target algorithm, “T” indicates the number of ties between IRBMO and the target algorithm, and “L” indicates the number of times the target algorithm outperformed IRBMO.

To further visually demonstrate the performance of the algorithms, we employ heatmaps to show the Friedman average ranks for each function, radar charts to display the algorithm ranks for each function, Sankey diagrams to illustrate the overall ranking distribution, stacked bar charts to count the occurrences of each algorithm at different ranks, line charts to present the Friedman average ranks, and convergence curves to compare the convergence speed of the algorithms. To ascertain statistical distinctions, the Wilcoxon rank-sum test was subsequently employed (significance level ℵ=0.05). This test performed pairwise comparisons between IRBMO and each competitor, with the detailed outcomes cataloged in Table S2. Within this table, the symbols “+”, “−”, and “=” are utilized to denote instances where IRBMO’s performance is significantly superior, significantly inferior, or statistically equivalent (no significant difference) to its rival, respectively. The final row provides an aggregate summary, tallying the total counts for these comparisons as “W” (Wins, “+”), “T” (Ties, “=”), and “L” (Losses, “−”). Finally, we utilize box plots to visualize and assess the convergence accuracy and stability of IRBMO.

All algorithms were executed under the same system configuration, which included a computer running the Windows 10 (64-bit) operating system, equipped with an Intel Xeon E-2224 3.40 GHz CPU and 16 GB of RAM. The experimental environment was established using MATLAB 2023a.

#### 4.1.2. Benchmark Functions

The empirical evaluation of the aforementioned algorithms was conducted using a benchmark suite composed of 41 problems in total, drawn from two distinct collections. Specifically, the CEC-2017 suite contributes 29 test functions, while the remaining 12 are from the CEC-2022 suite. The characteristics of each function are detailed in Table 2 and Table 3.

In the CEC-2017 benchmark, the functions F1 and F3 are unimodal, serving to assess the convergence efficiency of algorithms. Functions F4 through F10 are categorized as multimodal problems. Their primary purpose is to gauge an algorithm’s effectiveness in escaping local optima. Functions F11 to F20 are hybrid functions that incorporate rotation or translation, combining three or more benchmark functions from CEC-2017, with specific weights assigned to each sub-function. Functions F21 to F30 are composite problems, each formed by combining at least three distinct hybrid or CEC-2017 benchmarks that undergo rotational and shifting transformations; each sub-function has additional biases and weights, making optimization more challenging. In the CEC-2022 benchmark, F1 is classified as a unimodal function, while F2 through F5 are multimodal. Functions F6 to F8 are mixed, incorporating both unimodal and multimodal traits, and F9 to F12 are categorized as composite multimodal functions.

### 4.2. Strategies Effectiveness and Parameter Sensitivity Analysis

In this section, we present an ablation study alongside a parameter sensitivity analysis. We use the ablation study to confirm the contribution of the proposed strategies and employ the sensitivity analysis to investigate the key parameters.

#### 4.2.1. Performance Analysis on Ablation Experiments

To clearly validate the respective effectiveness and synergistic interaction of the three proposed improvement strategies, this section presents a series of ablation experiments. We compared the performance of the complete IRBMO algorithm against the following four degraded variants:

- **IRBMO-C**: Retains only the Logistic-Tent chaotic map.
- **IRBMO-B**: Retains only the dynamic balance factor.
- **IRBMO-JF**: Retains only the Jacobi curve and Lévy flight hybrid perturbation.
- **IRBMO-CB**: Retains the chaotic map and the balance factor.

All algorithms were evaluated on the 30-dimensional CEC-2017 test set, with experimental settings consistent with those in Section 4.1. Detailed results, including the mean (Mean), standard deviation (Std), Best, Worst and Wilcoxon rank-sum test outcomes (p=0.05), are summarized in Table S3.

The comparison results in Table S3 clearly indicate that the complete IRBMO algorithm significantly outperforms all four variants in terms of overall performance. Specifically, the single-strategy variants exhibit poor performance, demonstrating the limitations of any single improvement. Although IRBMO-JF performs better than other variants, it remains significantly inferior to the complete IRBMO algorithm integrating all three strategies.

The Wilcoxon test results further corroborate this finding, showing that IRBMO is significantly superior to all four variants on the vast majority of test functions. In summary, the ablation study strongly demonstrates that all three proposed strategies make a positive contribution to the algorithm’s performance, the absence of any single strategy leads to performance degradation, and that their combination produces a strong synergistic effect, collectively contributing to the superior performance of IRBMO.

#### 4.2.2. Sensitivity Analysis

In the IRBMO algorithm, the parameter $Rand_{6}$ from Section 3.4 controls the activation of the Jacobi curve perturbation (Equation (17)). To investigate the algorithm’s sensitivity to the probability threshold, and to validate the rationale for selecting a threshold of 0.05 in this study, we conducted a parameter sensitivity analysis. This experiment was also conducted on the 30-dimensional CEC-2017 test set, comparing the performance of IRBMO under four different threshold values (0.05, 0.1, 0.15, 0.2). All other experimental settings remained identical to those in Section 4.1. Table S3 presents the experimental results, which show that IRBMO achieves its optimal performance when the threshold is 0.05. As the threshold value increases, the algorithm’s performance progressively declines. This indicates that although the Jacobi curve perturbation assists the algorithm in escaping local optima, excessively frequent perturbations may disrupt stable convergence in the later stages of iteration, causing it to deviate from promising regions already identified. Therefore, the results confirm that 0.05 is a rational and effective threshold setting, as it strikes an optimal balance between maintaining population diversity and ensuring stable convergence. This setting is adopted for all experiments in this study.

### 4.3. Convergence Behavior Analysis

#### 4.3.1. Search Agent Distribution Analysis

**Figure 4:**
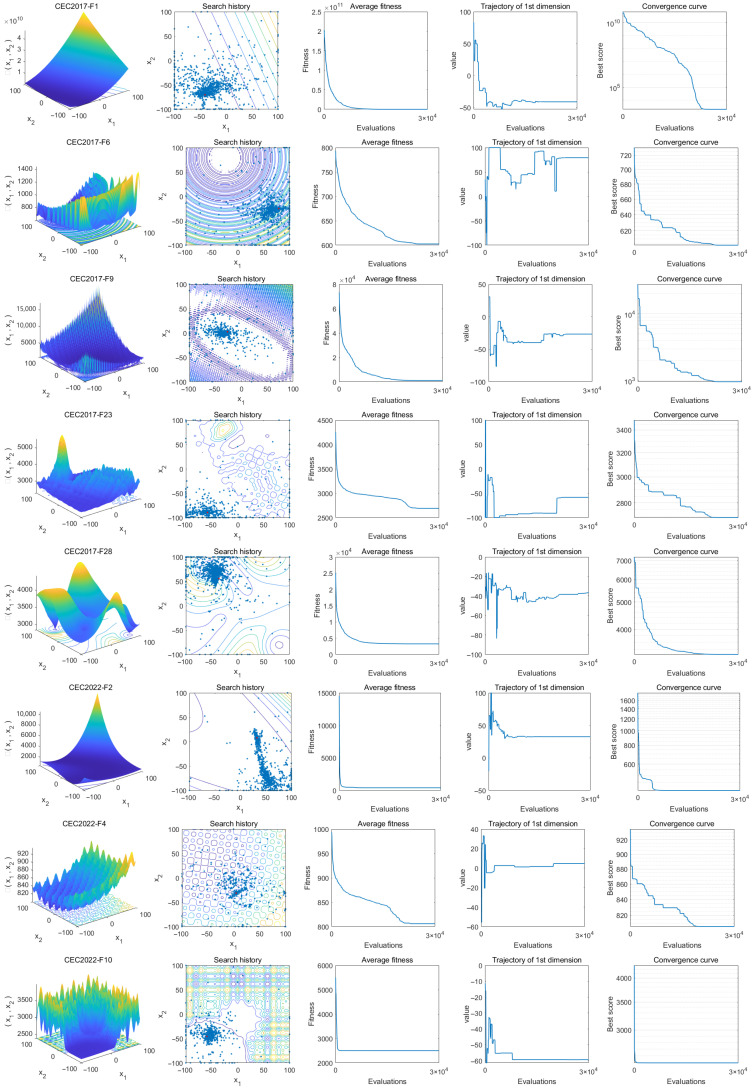
The convergence behavior of IRBMO.

In this part, we investigate the convergence characteristics of the IRBMO algorithm using five distinct subplots derived from experiments on the CEC-2017 and CEC-2022 benchmark datasets, as illustrated in Figure 4. The first subplot offers a 3D representation of the objective function’s landscape, allowing readers to perceive its complexity. In the second subplot, the agents’ search paths are depicted, highlighting their widespread dispersion throughout the landscape and underlining IRBMO’s robust global exploration ability. The third visualization tracks the evolution of the agents’ mean fitness values. At the outset, agents exhibit diverse distributions and fitness scores, which signifies a strong inclination for exploring the broader solution space. With ongoing iterations, this average fitness drops swiftly, implying that a majority of agents are converging towards finding the global optimum. The fourth subplot details the movement patterns of individual agents; their trajectories move from initial variability to convergence, showing a transition from exploratory global search to more focused local refinement—an approach that facilitates attainment of the optimal solution. Finally, the fifth subplot illustrates the IRBMO algorithm’s convergence curve. As the number of generations increases, the curve consistently trends downward, demonstrating the algorithm’s capacity to escape suboptimal local solutions and pursue even better global solutions.

#### 4.3.2. Exploration and Exploitation

**Figure 5:**
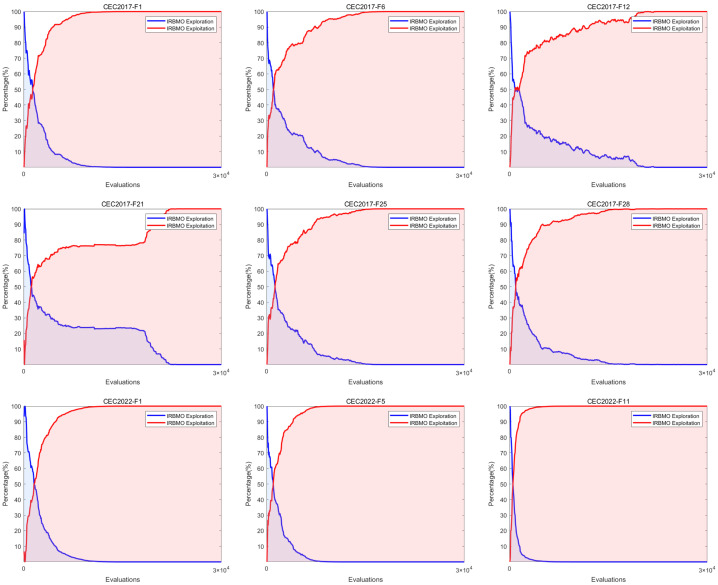
Balance between exploration and exploitation.

Meta-heuristic algorithms fundamentally rely on two essential processes: exploration and exploitation. The exploration phase enables thorough searching of the solution space, whereas the exploitation phase involves intensive searching within regions identified during exploration as potential candidates for the global optimum. A fundamental challenge in meta-heuristic design is establishing an equilibrium between exploration and exploitation. A successful balance is crucial, as it mitigates the algorithm’s risk of stagnating in local optima and aids in the effective identification of the global best solution. This, in turn, bolsters the algorithm’s robustness and adaptability. The exploration and exploitation behaviors are typically quantified using Equations (18) and (19). The performance of this balance, when tested on functions from the CEC-2017 and CEC-2022 suites, is depicted in Figure 5.

(18) $$Exploration\left(\%\right)=\frac{Di\nu (t)}{Di\nu_{max}}\times 100$$

(19) $$Exploitation\left(\%\right)=\frac{\left|Div\left(t\right)-Div_{max}\right|}{Div_{max}}\times 100$$

(20) $$Div\left(t\right)=\frac{1}{D}\sum_{d=1}^{D}\frac{1}{N}\sum_{i=1}^{N}\left|median\left(x_{d}\left(t\right)\right)-x_{id}\left(t\right)\right|$$

Figure 5 demonstrates that IRBMO emphasizes global exploration during the initial phase, before its focus gradually shifts toward local exploitation. Furthermore, the crossover point between the exploration and exploitation rates appears concentrated within the initial 10% of the iterative process. According to recent studies, the algorithm achieves optimal performance when exploration and exploitation constitute 10% and 90% of the search process, respectively, and are balanced [101]. This indicates that IRBMO demonstrates excellent exploration and convergence capabilities.

#### 4.3.3. Population Diversity Analysis

To further analyze the impact of the Jacobi–Lévy combination on the search process, we compared the population diversity of RBMO, IRBMO-CB, and IRBMO across various dimensions of the CEC-2017 and CEC-2022 test sets.

**Figure 6:**
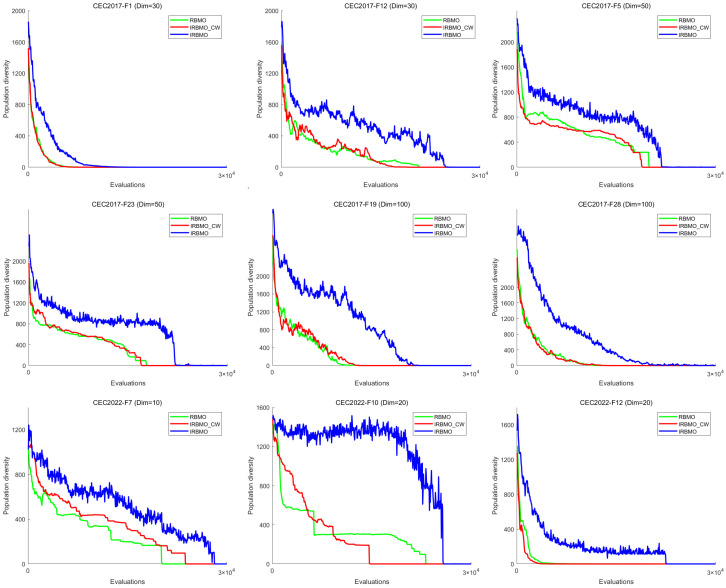
Population diversity of RBMO, IRBMO-CB and IRBMO.

Figure 6 illustrates the population diversity of the three algorithms on select functions across different dimensions. The figure clearly shows that the population diversity of RBMO and IRBMO-CB is comparable, whereas that of IRBMO is significantly higher. This indicates that the Jacobi–Lévy combination plays a significant role in enhancing population diversity. Furthermore, RBMO often converges prematurely in the middle stages of the search, a problem that IRBMO effectively mitigates. These results demonstrate that, compared to RBMO, IRBMO explores a broader search space more persistently, effectively mitigating premature convergence and the failure to escape local optima, highlighting the crucial role of the Jacobi–Lévy combination in this regard.

### 4.4. Performance Comparison on the CEC-2017 Test Suite

This section details the experimental results for 16 algorithms on the CEC-2017 test set, specifically for 30, 50, and 100 dimensions. Section 4.4.1 reports the number of wins achieved by IRBMO against the 15 competing algorithms across these dimensionalities. Section 4.4.2 provides two types of performance rankings—one based on mean values and another based on the Friedman test’s average rankings—accompanied by corresponding visualizations and analysis. Furthermore, Section 4.4.3 summarizes the Wilcoxon test results from Table S2, which compare IRBMO with the 15 other algorithms for each dimension. Finally, Section 4.4.4 utilizes box plots to illustrate the stability of all 16 algorithms.

#### 4.4.1. CEC-2017 Test Benchmark Functions Experimental Results

From Table S1, we can observe that IRBMO was compared 435 times against the average performance of 15 competing algorithms across each dimension of the CEC-2017 benchmark. As shown in the last row, IRBMO outperformed the competitors 413 times, 409 times, and 405 times in the 30, 50, and 100-dimensional cases, respectively, accounting for 94.94%, 94.02%, and 93.10% of the total comparisons. Compared to the classic RBMO algorithm, IRBMO won 28, 23, and 27 times in the three dimensions, respectively.

#### 4.4.2. Ranking of the CEC-2017 Test Set

In this section, we present the rankings of 16 algorithms on the CEC-2017 benchmark using radar charts, heatmaps, Sankey diagrams, stacked bar charts, and line graphs.

**Figure 7:**
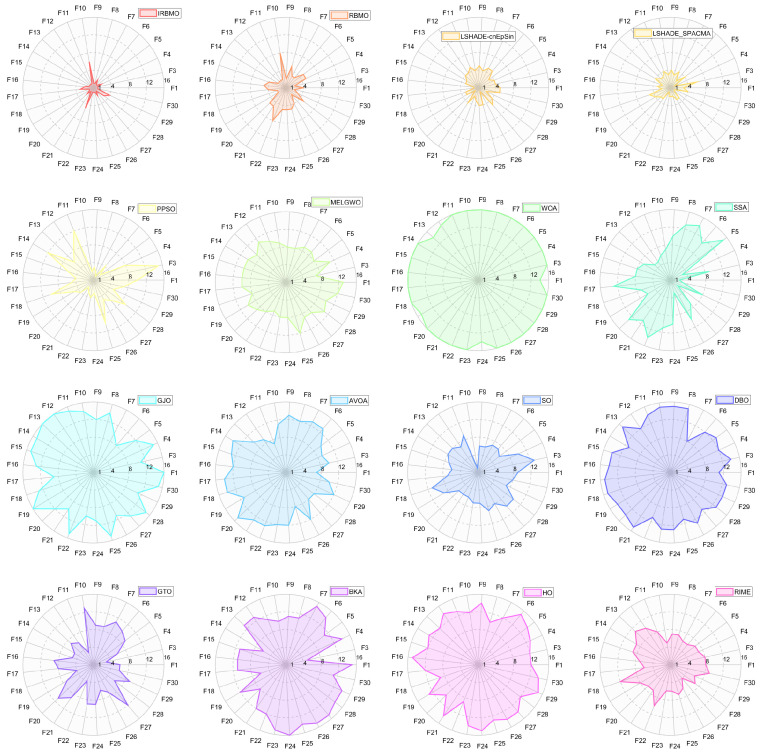
Comparison radar chart of the ranking of CEC-2017 benchmark (Dim = 30).

**Figure 8:**
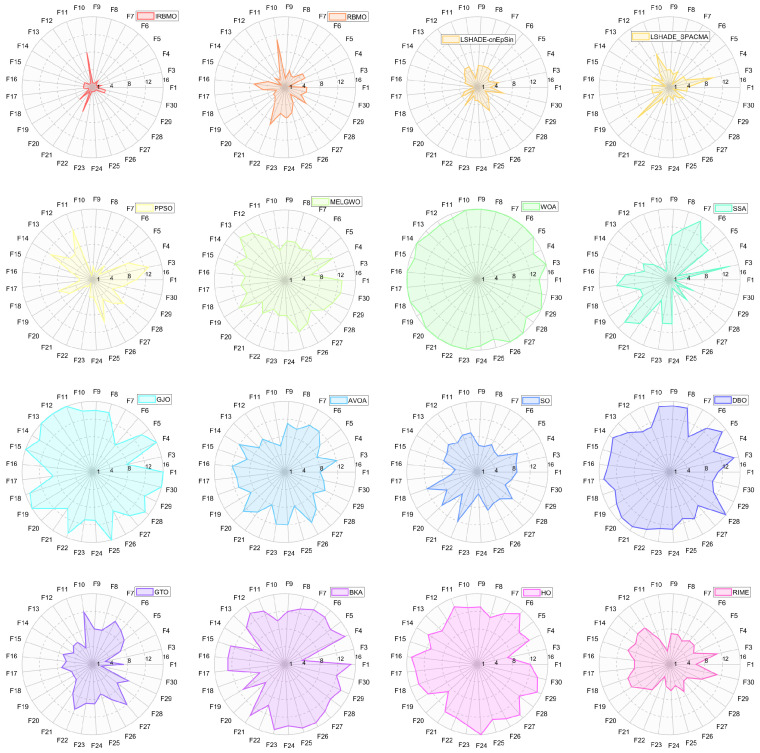
Comparison radar chart of the ranking of CEC-2017 benchmark (Dim = 50).

**Figure 9:**
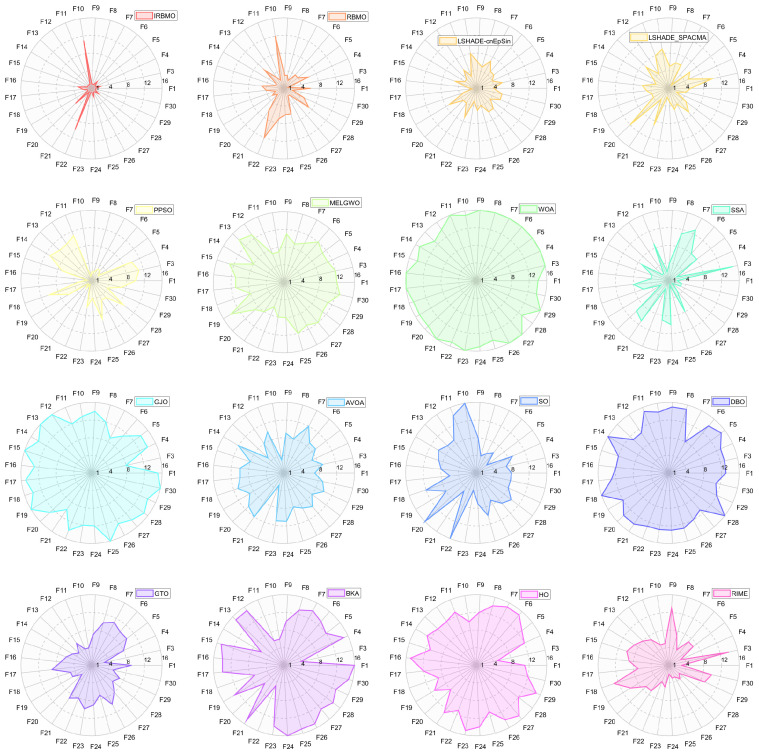
Comparison radar chart of the ranking of CEC-2017 benchmark (Dim = 100).

Figure 7, Figure 8 and Figure 9 display the radar charts of algorithm rankings. A smaller radar chart area indicates a higher overall ranking and stronger performance of the algorithm. Clearly, IRBMO exhibits the smallest radar chart areas in all three dimensions, demonstrating its superior overall ranking on the CEC-2017 benchmark.

**Figure 10:**
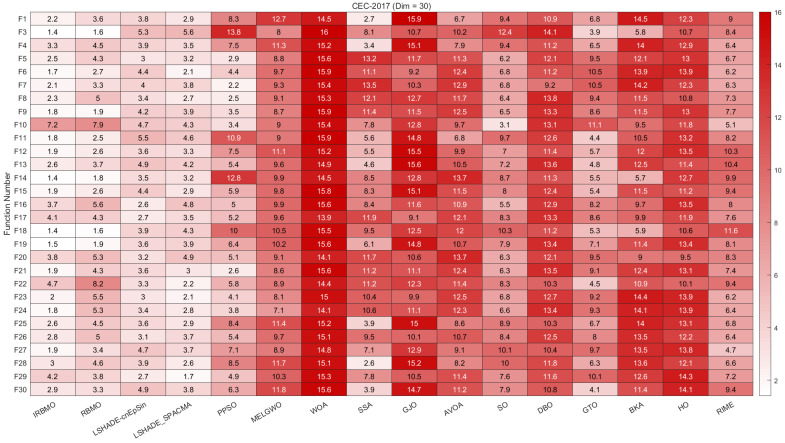
Comparison heatmap of the Friedman average ranking of CEC-2017 benchmark (Dim = 30).

**Figure 11:**
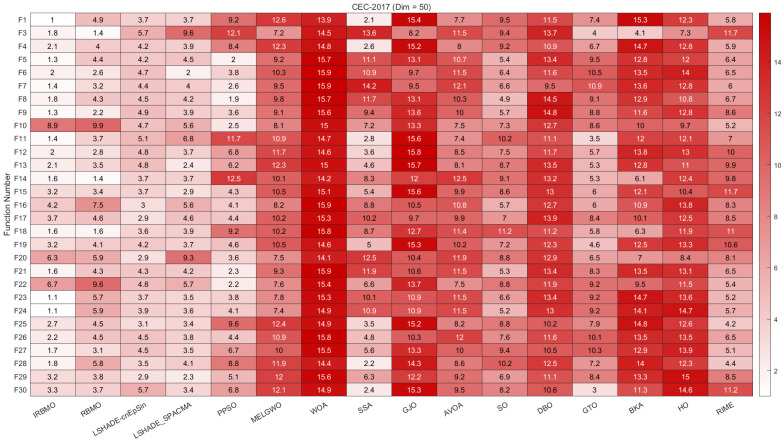
Comparison heatmap of the Friedman average ranking of CEC-2017 benchmark (Dim = 50).

**Figure 12:**
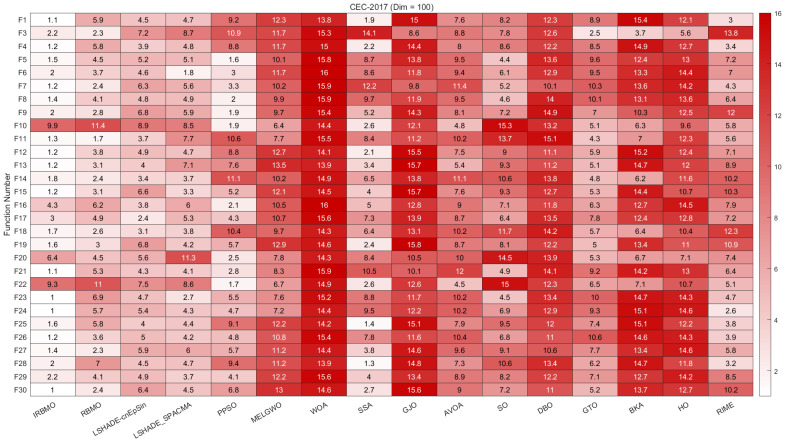
Comparison heatmap of the Friedman average ranking of CEC-2017 benchmark (Dim = 100).

Figure 10, Figure 11 and Figure 12 present heatmaps of the Friedman average rankings of 16 algorithms across different dimensions. The heatmaps show the average ranking of each algorithm for each function. A color closer to white indicates a higher ranking and better performance, while a color closer to red indicates a lower ranking and poorer performance. The performance of IRBMO on various types of functions is as follows:

- **Unimodal Functions (F1 and F3):** The primary purpose of unimodal functions is to assess the convergence speed of the algorithms. The heatmap indicates that for F1, IRBMO consistently achieves the best Friedman average ranking across all dimensions. For F3, IRBMO obtains the best ranking in 30 and 100 dimensions, while in 50 dimensions, its convergence speed is second only to RBMO. This demonstrates that, in most cases, IRBMO outperforms RBMO and other competing algorithms in terms of convergence speed.
- **Multimodal functions (F4–F10):** This category of functions contains multiple local optima, and their primary role is to evaluate an algorithm’s ability to escape from local optima. For F10, the average rankings of IRBMO across different dimensions are 7.2, 8.9, and 9.9, indicating that IRBMO performs poorly on this function. Observations of the classical RBMO reveal average rankings of 7.9, 9.9, and 11.4 across the same dimensions. Clearly, the decline in convergence accuracy with increasing dimensionality has been a persistent issue since the classical RBMO algorithm. Although IRBMO provides slight improvements, it does not completely resolve this problem. Apart from F10, IRBMO maintains average rankings below 3 across all dimensions for the other multimodal functions, demonstrating its capability to escape from local optima.
- **Hybrid functions (F11–F20):** For these 10 functions, when the dimension is 30, IRBMO achieves the highest average ranking in all functions except for F16, F17, and F20. When the dimension is 50, IRBMO ranks first in F11, F12, F13, F18, and F19. For the 100-dimensional case, IRBMO ranks first in all functions except for F16, F17, and F20. Additionally, it is evident that in most cases, IRBMO maintains an average ranking below 4, with the exception of F20 at dimensions 50 and 100, where its performance is suboptimal.
- **Composition functions (F21–F30):** In the most complex composition functions, IRBMO also maintains an average ranking below 4. When the dimension is 30, IRBMO achieves the highest average ranking in all functions except for F22, F28, and F29. When the dimension is 50, IRBMO ranks first in all functions except for F22, F29, and F30. For the 100-dimensional case, IRBMO ranks first in all functions except for F22, F25, and F28.

The above analysis indicates that, on the CEC-2017 benchmark suite, IRBMO demonstrates good convergence speed, the ability to escape from local optima, and the potential to solve high-dimensional complex problems.

**Figure 13:**
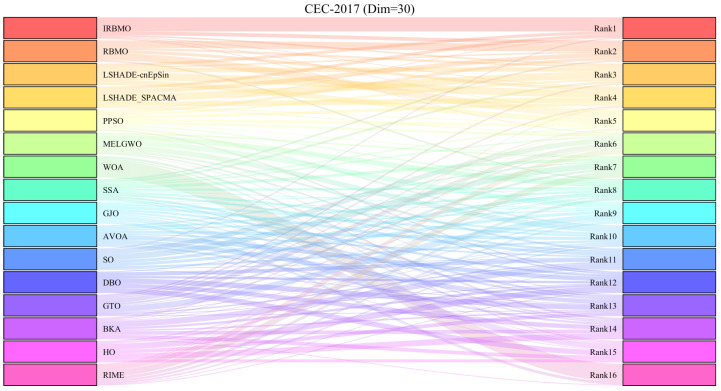
Sankey diagram of the ranking of CEC-2017 benchmark (Dim = 30).

**Figure 14:**
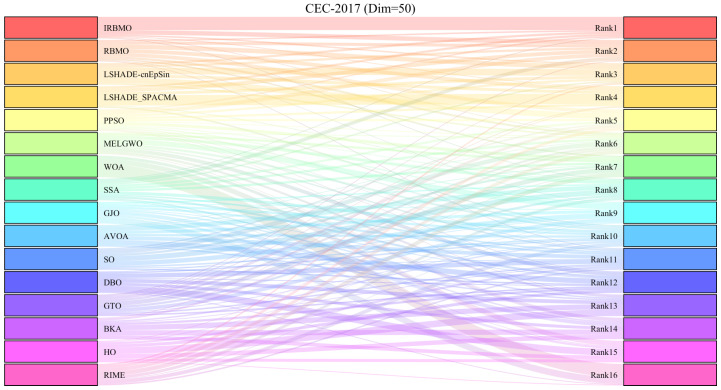
Sankey diagram of the ranking of CEC-2017 benchmark (Dim = 50).

**Figure 15:**
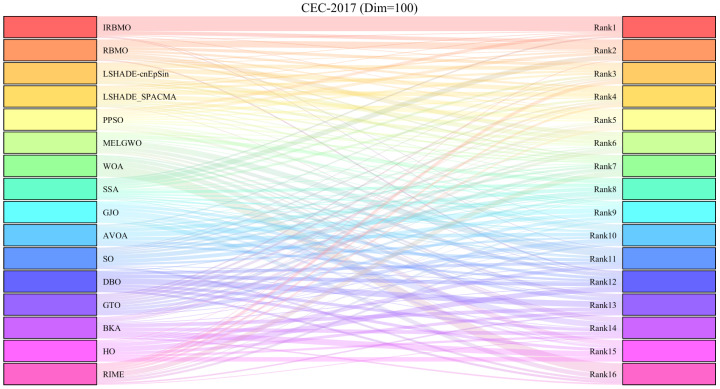
Sankey diagram of the ranking of CEC-2017 benchmark (Dim = 100).

Figure 13, Figure 14 and Figure 15 present the Sankey diagrams illustrating the performance rankings of 16 algorithms on various test functions of the CEC-2017 benchmark suite at dimensions 30, 50, and 100. It is evident that, across all dimensions, IRBMO has the thickest lines connecting to the “Rank 1”, indicating that IRBMO achieves the most first-place rankings in the CEC-2017 test suite.

**Figure 16:**
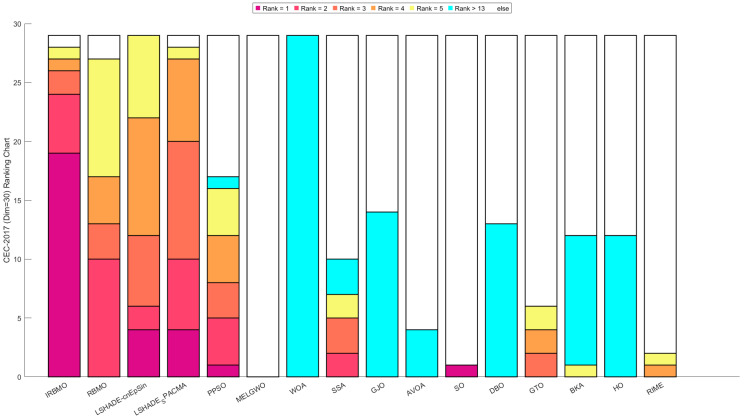
Stacked bar chart of the ranking of CEC-2017 benchmark (Dim = 30).

**Figure 17:**
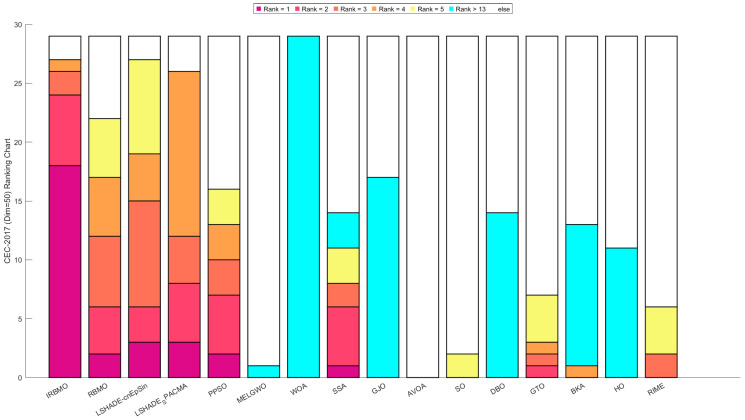
Stacked bar chart of the ranking of CEC-2017 benchmark (Dim = 50).

**Figure 18:**
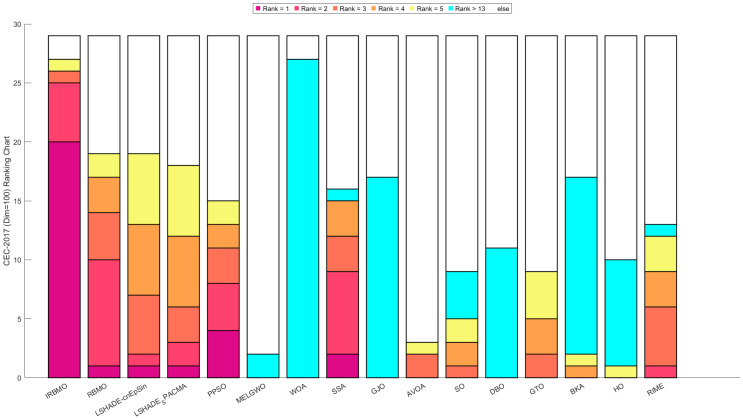
Stacked bar chart of the ranking of CEC-2017 benchmark (Dim = 100).

Figure 16, Figure 17 and Figure 18 show stacked bar charts representing algorithm rankings. In these figures, the purple section indicates the number of first-place rankings obtained by each algorithm. Clearly, IRBMO consistently achieves the highest number of first-place rankings, with 19, 18, and 20 first-place finishes at dimensions 30, 50, and 100, respectively. The blue-shaded area corresponds to the count of instances where an algorithm performed among the three worst solutions. Notably, in the 29 test functions of the CEC-2017 benchmark, WOA ranks in the bottom three in the majority of the functions. Additionally, GJO, DBO, BKA, and HO also rank poorly in a significant number of functions, suggesting that these algorithms perform poorly on the CEC-2017 test suite. In contrast, RBMO, LSHADE-cnEpSin, and LSHADE_SPACMA, although not outperforming IRBMO overall, consistently rank in the top five in most functions and never appear in the bottom three, indicating strong overall performance on the CEC-2017 benchmark.

**Figure 19:**
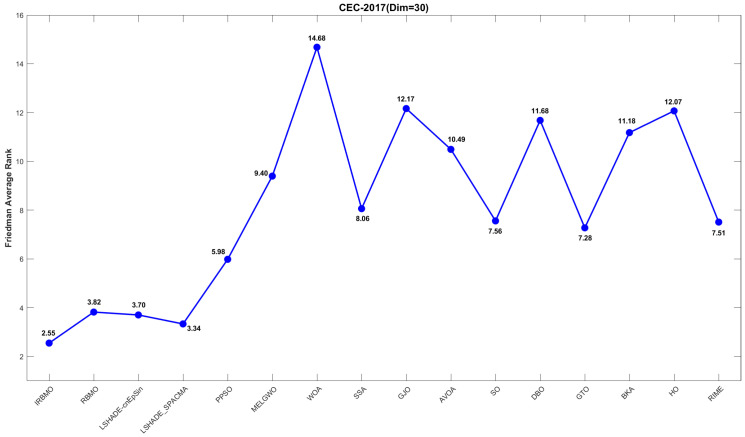
Friedman average ranking line charts of the CEC-2017 (Dim = 30).

**Figure 20:**
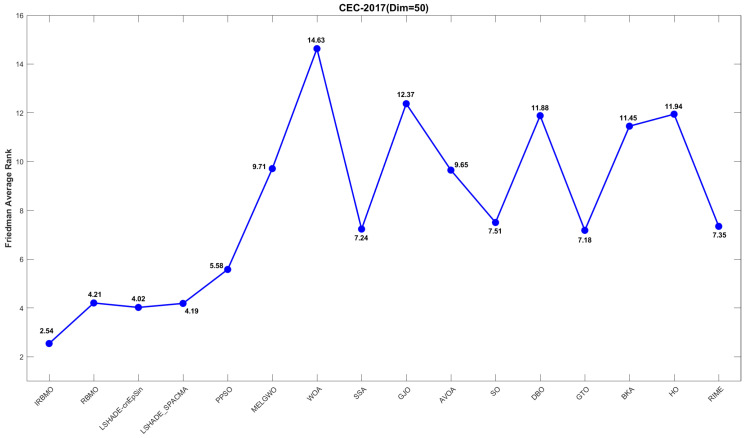
Friedman average ranking line charts of the CEC-2017 (Dim = 50).

**Figure 21:**
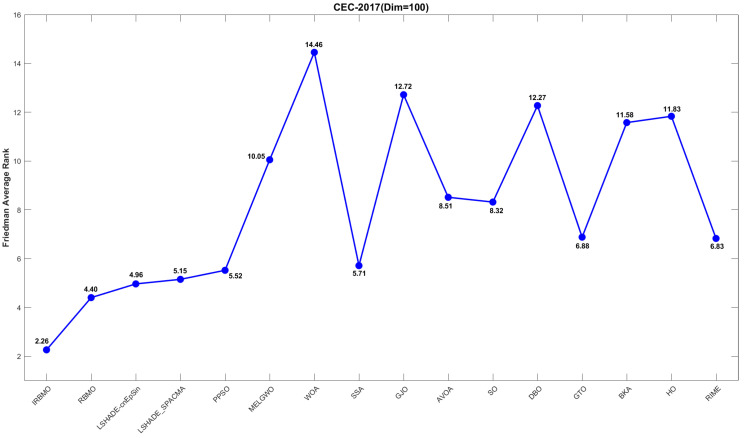
Friedman average ranking line charts of the CEC-2017 (Dim = 100).

Figure 19, Figure 20 and Figure 21 display the Friedman average ranking line plots for the CEC-2017 benchmark suite. It is evident from the figures that IRBMO consistently maintains the first position across all dimensions. Notably, the average rankings for IRBMO at dimensions 30, 50, and 100 are 2.55, 2.54, and 2.26, respectively, while the second-place rankings are 3.34, 4.02, and 4.40. The difference in average rankings between IRBMO and the second-place algorithm is 0.79, 1.48, and 2.14, respectively, across the three dimensions. Clearly, as the dimensionality increases, the advantage of IRBMO over the competing algorithms becomes more pronounced, indicating the strong potential of IRBMO for solving high-dimensional problems.

**Figure 22:**
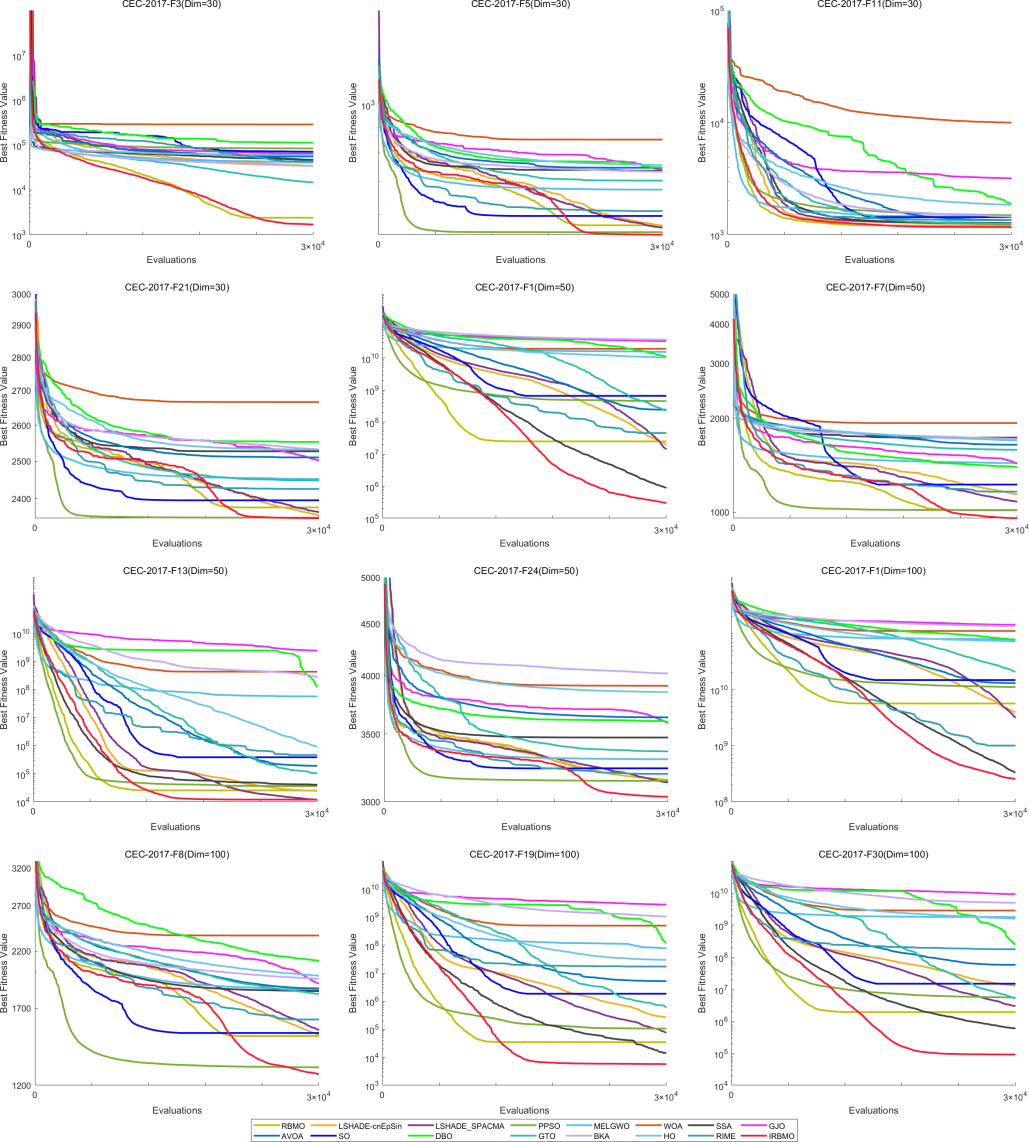
CEC-2017 test function convergence curve.

A comparative illustration of the convergence trajectories for 16 algorithms is provided in Figure 22, covering selected CEC-2017 functions across multiple dimensions. The figure shows that, for the unimodal functions F3 (Dim = 30), F1 (Dim = 50), and F1 (Dim = 100), IRBMO quickly converges to a solution close to the global optimum early in the iteration, indicating its strong exploration capability. For the multimodal functions F5 (Dim = 30), F7 (Dim = 50), and F8 (Dim = 100), other algorithms tend to get trapped in local optima in the later stages of the search, whereas HTSO maintains particle diversity and continues to explore better solutions, thus maintaining good convergence accuracy. For the hybrid functions F11 (Dim = 30), F13 (Dim = 50), F19 (Dim = 100) and the composition functions F21 (Dim = 30), F24 (Dim = 50), F30 (Dim = 100), IRBMO continues to converge rapidly in the later stages of the search while retaining the ability to discover new local optima. This suggests that the introduction of the Jacobi mutation strategy during the development phase effectively helps particles escape from local optima and explore previously unsearched regions, thereby improving convergence accuracy.

#### 4.4.3. Wilcoxon Rank Sum Test of the CEC-2017 Test Set

Table S2 presents the Wilcoxon test results across different dimensions of the CEC-2017 benchmark suite. As shown, the number of cases where IRBMO exhibits significant differences from the compared algorithms is 383, 377, and 395 for the 30-, 50-, and 100-dimensional problems, accounting for 88.05%, 86.67%, and 90.80% of all comparisons, respectively.

It is worth noting that the Wilcoxon test results for the 30-, 50-, and 100-dimensional cases indicate that IRBMO significantly outperforms the classical RBMO on 17, 17, and 24 functions, respectively, with zero losses against RBMO. Clearly, IRBMO demonstrates statistically significant performance differences compared to other competing algorithms, including RBMO, on the CEC-2017 benchmark suite, which confirms the effectiveness of the proposed improvements.

#### 4.4.4. Box Plot of the CEC-2017 Test Set

**Figure 23:**
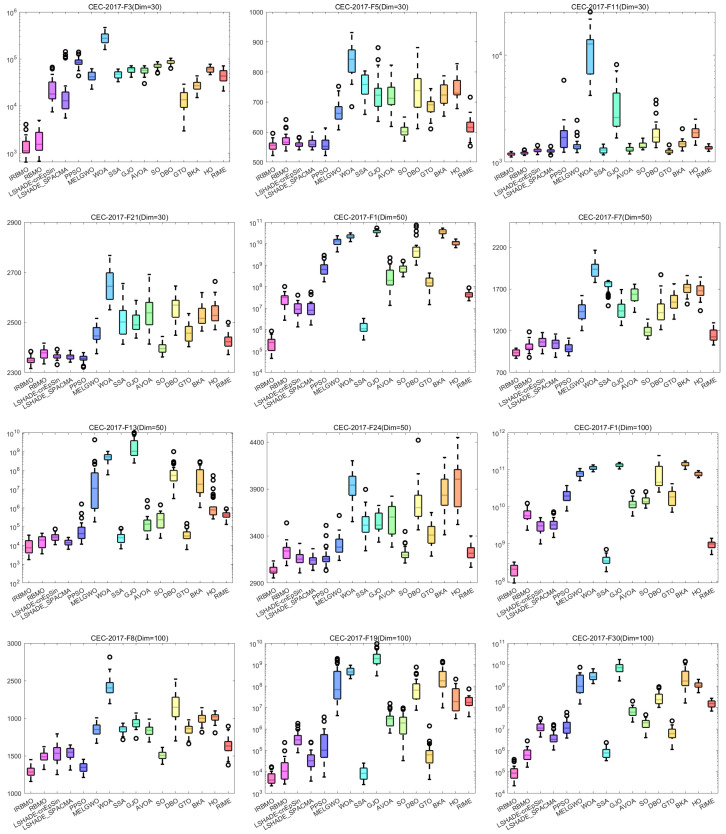
CEC-2017 test function boxplots.

Figure 23 presents boxplots of the results from 16 algorithms independently run 30 times, including the functions F3, F5, F11, and F21 at 30 dimensions, F1, F7, F13, and F24 at 50 dimensions, and F1, F8, F19, and F30 at 100 dimensions. It is important to note that some of the plots use a logarithmic scale on the y-axis, meaning that algorithms with stronger stability and higher convergence accuracy may appear to have wider boxes. From the figure, it can be observed that IRBMO has the narrowest boxes on F3 and F11 at 30 dimensions, F1, F7, F24 at 50 dimensions, and F19 and F30 at 100 dimensions. Furthermore, IRBMO shows no outliers on F11 at 30 dimensions, F7, F13, and F24 at 50 dimensions, and F1 and F8 at 100 dimensions. Notably, on F11 at 30 dimensions, most algorithms, including the CEC competition-winning algorithms, exhibit outliers, whereas IRBMO maintains a narrow box without any outliers, demonstrating both superior convergence accuracy and stability.

These functions cover three dimensions of the CEC-2017 benchmark suite and include unimodal, multimodal, hybrid, and composition functions, representing various function types. Based on the experimental findings, IRBMO delivers a compelling balance of high convergence accuracy, exceptional stability, and robust performance.

### 4.5. Performance Comparison on the CEC-2022 Test Suite

This section presents the empirical results of 16 algorithms on the CEC-2022 test set for 10 and 20 dimensions. Section 4.5.1 reports the number of wins achieved by IRBMO against the 15 competing algorithms. Section 4.5.2 presents rankings of the 16 algorithms based on their mean values, along with various visualizations and analyses derived from the Friedman test’s average rankings. Section 4.5.3 summarizes the data from Table S2, highlighting the Wilcoxon test results that compare IRBMO with each competing algorithm. Finally, Section 4.5.4 employs box plots to compare the stability of all 16 algorithms.

#### 4.5.1. CEC-2022 Test Benchmark Functions Experimental Results

Table S1 reports the experimental results on the 10- and 20-dimensional instances of the CEC-2022 benchmark suite. Across both dimensions, IRBMO was compared with competing algorithms in a total of 180 pairwise tests, achieving 165 wins at 10 dimensions and 164 wins at 20 dimensions, corresponding to 91.67% and 91.11% of all comparisons, respectively. Specifically, when compared with RBMO, IRBMO achieved 10 wins and 2 losses at 10 dimensions, and 9 wins and 3 losses at 20 dimensions. In contrast, against LSHADE-cnEpSin, IRBMO won only 5 times and lost 7 times at 10 dimensions, suggesting that IRBMO underperforms LSHADE-cnEpSin on lower-dimensional problems. However, as the dimensionality increases to 20, IRBMO secures 8 wins versus 4 losses, successfully surpassing LSHADE-cnEpSin. IRBMO’s efficacy in managing high-dimensional complex optimization problems is therefore underscored by this result.

#### 4.5.2. Ranking of the CEC-2022 Test Set

**Figure 24:**
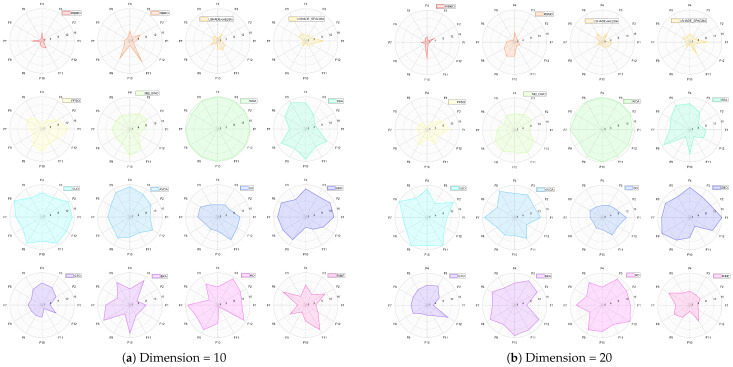
Comparison radar charts of the ranking of CEC-2022 benchmark.

**Figure 25:**
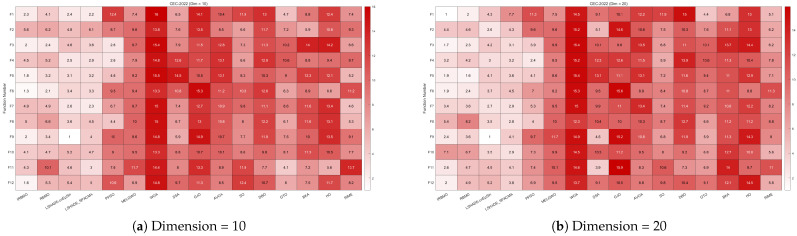
Comparison heatmaps of the Friedman average ranking of CEC-2022 benchmark.

Figure 24 presents a radar chart displaying the performance rankings of 16 algorithms on the CEC-2022 benchmark suite at 10 and 20 dimensions. From the figure, it is evident that the areas corresponding to IRBMO and LSHADE-cnEpSin are notably smaller than those of the other algorithms. Figure 25 shows the heatmap of the Friedman average rankings for the 16 algorithms. At 10 dimensions, IRBMO outperforms the others on F3, F5, F6, and F12, achieving the top average ranking. At 20 dimensions, IRBMO ranks first on F1, F3, F6, F11, and F12.

**Figure 26:**
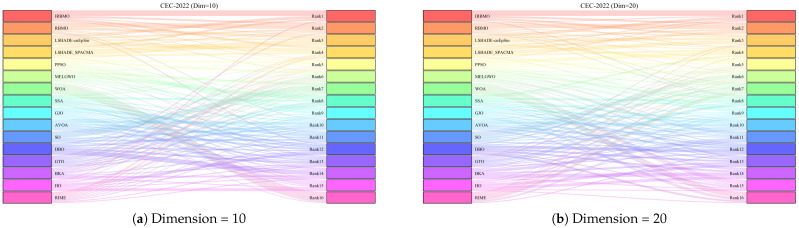
Sankey diagrams of the ranking of CEC-2022 benchmark.

**Figure 27:**
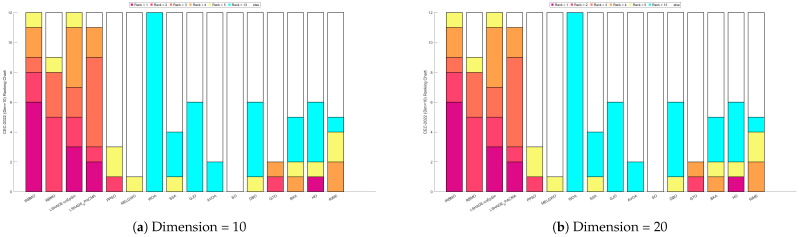
Stacked bar charts of the ranking of CEC-2022 benchmark.

Figure 26 presents the Sankey diagram depicting the performance rankings of 16 algorithms on various test functions in the CEC-2022 benchmark suite at 10 and 20 dimensions. It is evident that, across all dimensions, IRBMO consistently maintains the thickest connections to Rank 1. Figure 27 displays a stacked bar chart, where the purple color indicates the number of times each algorithm achieved first place. Both the Sankey diagram and the stacked bar chart clearly demonstrate that IRBMO secured the most first-place rankings. Additionally, we observe that IRBMO achieved a top-5 ranking in all test functions at 10 dimensions, whereas at 20 dimensions, one of the test functions did not make the top 5, indicating slightly inferior performance compared to LSHADE-cnEpSin. However, IRBMO’s higher number of first-place rankings indicates a competitive edge over LSHADE-cnEpSin in certain aspects. The blue sections represent the number of functions where the algorithms ranked in the bottom three. It is apparent from the blue sections that WOA, SSA, GJO, AVOA, DBO, BKA, HO, and RIME performed poorly on the CEC-2022 benchmark suite.

**Figure 28:**
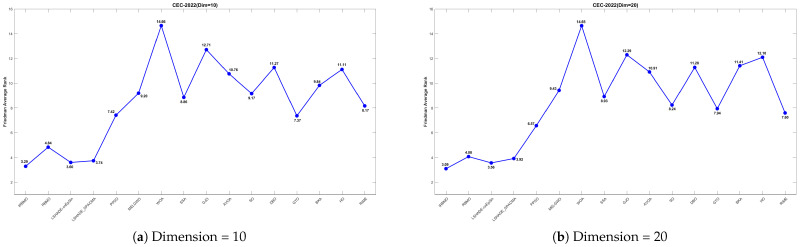
Friedman average ranking line charts of the CEC-2022.

Figure 28 shows the line chart of the Friedman average rankings for the CEC-2022 benchmark suite at 10 and 20 dimensions. From the figure, it is evident that IRBMO ranks first at 10 dimensions with an average ranking of 3.29. LSHADE-cnEpSin follows in second place, with an average ranking of 3.60, a difference of 0.31 compared to IRBMO. At 20 dimensions, IRBMO again ranks first with an average ranking of 3.09. LSHADE-cnEpSin remains in second place, with an average ranking of 3.56, a difference of 0.47 from IRBMO. Compared to 10 dimensions, the gap between IRBMO and the second-place algorithm at 20 dimensions has increased by 0.16. This result aligns with the findings from the CEC-2017 test suite, further validating IRBMO’s capability in solving high-dimensional optimization problems.

**Figure 29:**
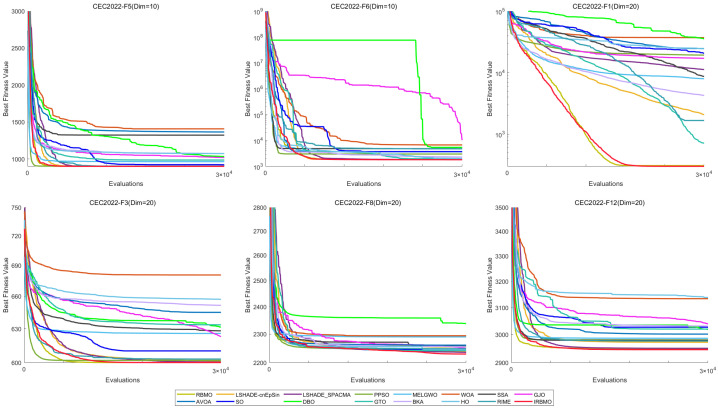
CEC-2022 test function boxplots.

Figure 29 illustrates the convergence curves of IRBMO and 15 benchmark algorithms. IRBMO decisively outperforms the competing methods, achieving both rapid convergence and superior precision in its results.

#### 4.5.3. Wilcoxon Rank Sum Test of the CEC-2022 Test Set

Table S2 presents the results of the Wilcoxon test. IRBMO outperforms the comparison algorithms significantly in 143 and 152 cases at the two dimensions, accounting for 79.44% and 84.44% of the total tests, respectively. Notably, the Wilcoxon test results at 10 and 20 dimensions indicate that, across 12 test functions, IRBMO performs significantly better than the classic RBMO in 5 and 6 functions, respectively, while RBMO shows significantly better performance than IRBMO in none of the functions. This suggests that the improvement strategy of IRBMO is effective in enhancing convergence accuracy on the CEC-2022 test suite. Moreover, the Wilcoxon test results show that at 10 dimensions, IRBMO outperforms LSHADE-cnEpSin in three functions and performs worse in three functions. This indicates that, although the experimental results in Section 4.5.2 show that LSHADE-cnEpSin wins more frequently than IRBMO at 10 dimensions, its performance is not significantly superior to IRBMO.

#### 4.5.4. Box Plot of the CEC-2022 Test Set

**Figure 30:**
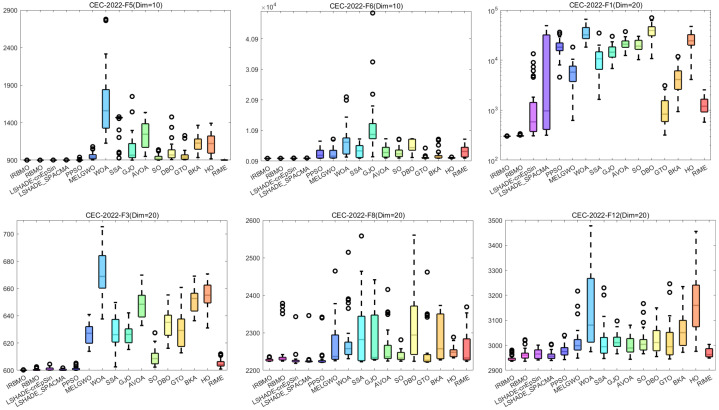
CEC-2022 test function convergence curve.

The statistical distribution of experimental results for 16 algorithms is depicted via boxplots in Figure 30, utilizing selected, diverse functions from the CEC-2022 test set. It is evident that the boxes for IRBMO are consistently narrower, indicating that IRBMO demonstrates stronger stability compared with other algorithms. Furthermore, IRBMO consistently converges near the global optimum, suggesting that it not only maintains high stability but also achieves superior convergence accuracy.

### 4.6. Summary of the CEC Test Set Experiment

In this section, the efficacy of the proposed IRBMO algorithm is comprehensively evaluated using 41 test functions sourced from the CEC-2017 and CEC-2022 test suites. The experimental results demonstrate that IRBMO significantly outperforms the other metaheuristic algorithms compared across both test suites. Compared to the classic RBMO algorithm, IRBMO exhibits stronger stability, higher convergence accuracy, and faster convergence speed, particularly on high-dimensional complex functions where IRBMO often shows superior performance. These results suggest that the improvements made to IRBMO address the shortcomings of RBMO effectively.

## 5. Engineering Design Problems

### 5.1. Real-World Constrained Optimization Problems

In Section 4, we evaluated the performance of the IRBMO algorithm under different dimensions of multiple CEC test sets. Next, we applied the IRBMO to four engineering design problems to assess its ability to solve real-world Constrained Optimization Problems(COPs). Table 4 presents the basic information of these four real-world COPs, which are roughly sorted according to the problem dimensions and the complexity of the constraint conditions. All the problems are sourced from the CEC-2020 competition [102]. All comparison algorithms shared a standardized configuration, where the population size was fixed at 30 and the maximum number of fitness evaluations was capped at 30,000. Subsequently, 10 independent trials were performed for each algorithm. The Best and Mean values were recorded for comparison, and the variable values corresponding to the Best were also provided. Finally, the rankings of the algorithms are determined based on the Mean values.

Here, *D* represents the dimension of the problem, $N_{g}$ denotes the number of inequality constraints, and $N_{h}$ stands for the number of equality constraints.

### 5.2. Constraint Handling Technique

Since IRBMO is an unconstrained optimization algorithm, this study employs the penalty function method to transform the constrained optimization problem into an unconstrained one for the experiments. The fitness function *F* for the resulting unconstrained problem is defined, where $\vec{x}$ represents the solution vector; $g_{i}$ and $h_{j}$ denote the inequality and equality constraints, respectively; $N_{g}$ and $N_{h}$ are the number of inequality and equality constraints; and $\zeta_{i}$ and $\xi_{j}$ are their corresponding penalty factors.

(21) $$F\left(\vec{x},\zeta_{i},\xi_{j}\right)=f\left(\vec{x}\right)+\sum_{i=1}^{N_{g}}\zeta_{i}g_{i}^{2}\left(\vec{x}\right)+\sum_{j=1}^{N_{h}}\xi_{j}h_{j}^{2}\left(\vec{x}\right)$$

(22) $$\left\{\begin{array}{cc} g_{i}\left(\vec{x}\right)\leq 0\;, & \;i=1,...,N_{g}\\ |h_{j}(\vec{x})|\leq \varepsilon \;, & \;j=1,...,N_{h} \end{array}\right.$$

Equation (22) is the general expression for the inequality and equality constraints where $\varepsilon =10^{-6}$ is the tolerance threshold for constraint violation. If the constraints $g_{i}\left(\vec{x}\right)$ and $h_{j}\left(\vec{x}\right)$ satisfy Equation (22), the corresponding penalty term is 0. Conversely, if they do not satisfy Equation (22), their actual calculated values are multiplied by penalty factors to generate a significant penalty for the constraint violation. The algorithm is considered to have found a feasible solution if all constraints satisfy.

In this method, solutions that violate the constraints are assigned large penalty factors. This results in infeasible solutions having a significantly higher fitness than feasible solutions, ultimately guiding the population toward the feasible region.

### 5.3. Tension/Compression Spring Design (TCPD (Case1))

For this optimization problem, the coil’s weight is designated as the objective function to be minimized, while the design’s feasibility is dictated by three specific engineering constraints. The problem can be represented mathematically as follows:

(23) $$\begin{array}{cc} \; & Minimize:\\ \; & f\left(\vec{x}\right)=x_{1}^{2}x_{2}\left(2+x_{3}\right)\\ \; & subject\;to:\\ \; & g_{1}\left(\vec{x}\right)=1-\frac{x_{2}^{3}x_{3}}{71785x_{1}^{4}}\leq 0\\ \; & g_{2}\left(\vec{x}\right)=\frac{4x_{2}^{2}-x_{1}x_{2}}{12566(x_{2}x_{1}^{3}-x_{1}^{4})}+\frac{1}{5108x_{1}^{2}}-1\leq 0\\ \; & g_{3}\left(\vec{x}\right)=1-\frac{140.45x_{1}}{x_{2}^{2}x_{3}}\leq 0\\ \; & g_{4}\left(\vec{x}\right)=\frac{x_{1}+x_{2}}{1.5}-1\leq 0\\ \; & with\;bounds:\\ \; & 0.05\leq x_{1}\leq 2.00\\ \; & 0.25\leq x_{2}\leq 1.30\\ \; & 2.00\leq x_{3}\leq 15.0 \end{array}$$

where $x_{1}$ is the wire diameter, $x_{2}$ is the coil diameter, and $x_{3}$ is the number of coils. Table 5 presents the performance results of IRBMO and its comparison algorithms. On this problem, IRBMO achieved a mean value of 1.2665 × 10^2^, ranking first among all compared algorithms. The next best-performing algorithm was RBMO, with a mean value of 1.2666 × 10^2^. The Wilcoxon signed-rank test results indicate that there is no significant difference between the performance of IRBMO and that of LSHADE-cnEpSin, LSHADE, and DE on this problem. However, IRBMO significantly outperformed the remaining seven algorithms.

### 5.4. Step-Cone Pulley Problem (SCP)

This problem addresses the optimal design of a 4 step-cone pulley by minimizing its weight. The design process utilizes five variables—four defining the stage diameters and one for the width—and is subject to 11 nonlinear constraints mandating a transmission power of 0.75 hp. Mathematically, the problem can be formulated as follows.

(24) $$\begin{array}{cc} \; & Minimize:\\ \; & f\left(\vec{x}\right)=\rho \omega \left[d_{1}^{2}\left\{11+{\left(\frac{N_{1}}{N}\right)}^{2}\right\}+d_{2}^{2}\left\{1+{\left(\frac{N_{2}}{N}\right)}^{2}\right\}+d_{3}^{2}\left\{1+{\left(\frac{N_{3}}{N}\right)}^{2}\right\}+d_{4}^{2}\left\{1+{\left(\frac{N_{4}}{N}\right)}^{2}\right\}\right]\\ \; & subject\;to:\\ \; & h_{1}\left(\vec{x}\right)=C_{1}-C_{2}=0\\ \; & h_{2}\left(\vec{x}\right)=C_{1}-C_{3}=0\\ \; & h_{3}\left(\vec{x}\right)=C_{1}-C_{4}=0\\ \; & g_{i=1,2,3,4}\left(\vec{x}\right)=-R_{i}\leq 2\\ \; & g_{i=5,6,7,8}\left(\vec{x}\right)=\left(0.75\times 745.6998\right)-P_{i}\leq 0\\ \; & where:\\ \; & C_{i}=\frac{\pi d_{i}}{2}\left(1+\frac{N_{i}}{N}\right)+\frac{{\left(\frac{N_{i}}{N}-1\right)}^{2}}{4a}+2a,\;i=\left(1,\;2,\;3,\;4\right)\\ \; & R_{i}=\exp\left(\mu \left\{\pi -2sin^{-1}\{\left(\frac{N_{i}}{N}-1\right)\frac{d_{i}}{2a}\right\}\}\right),\;i=\left(1,\;2,\;3,\;4\right)\\ \; & P_{i}=st\omega \left(1-R_{i}\right)\frac{\pi d_{i}N_{i}}{60},\;i=\left(1,\;2,\;3,\;4\right)\\ \; & t=8\mathrm{mm},\;s=1.75\mathrm{MPa},\;\mu =0.35,\;\rho =7200\mathrm{kg}/m^{3},\;a=3\mathrm{mm},\\ \; & N=350,\;N_{1}=750,\;N_{2}=450,\;N_{3}=250,\;N_{4}=150. \end{array}$$

where $d_{1}$, $d_{2}$, $d_{3}$ and $d_{4}$ are the respective pulley diameters, and ω is the pulley width. Table 6 presents the experimental results for the Step-Cone Pulley problem. From the perspective of the mean value, IRBMO ranked first among all algorithms, achieving an average result of 1.6070 × 10^1^. According to the results of the Wilcoxon signed-rank test, the performance of IRBMO was significantly better than that of all compared algorithms except LSHADE_SPACMA. The discrepancy between the mean value and the Wilcoxon test suggests that, although LSHADE_SPACMA outperformed IRBMO in most runs, IRBMO occasionally produced exceptionally good results. Additionally, as shown in the table, GJO fails to converge on this problem and performs poorly.

### 5.5. 10-Bar Truss Design (10-BT)

The optimization target for this truss structure involves minimizing its weight. This design is governed by specific frequency constraints, and the complete mathematical model is defined as follows:

(25) $$\begin{array}{cc} \; & Minimize:\\ \; & f\left(\vec{x}\right)=\sum_{i=1}^{10}L_{i}\left(x_{i}\right)\rho_{i}A_{i}\\ \; & subject\;to:\\ \; & g_{1}\left(\vec{x}\right)=\frac{7}{\omega_{1}\left(\vec{x}\right)}-1\leq 0\\ \; & g_{2}\left(\vec{x}\right)=\frac{15}{\omega_{2}\left(\vec{x}\right)}-1\leq 0\\ \; & g_{3}\left(\vec{x}\right)=\frac{20}{\omega_{3}\left(\vec{x}\right)}-1\leq 0\\ \; & with\;bounds:\\ \; & 6.45\times 10^{-5}\leq A_{i}\leq 5\times 10^{-3},\;i=1,\;2,\;\ldots ,\;10.\\ \; & where:\\ \; & \vec{x}=\left\{A_{1},\;A_{2},\;\ldots ,\;A_{10}\right\},\;\rho =2770. \end{array}$$

Here, $A_{1}$ through $A_{1}0$ denote the cross-sectional areas of the ten members, respectively. The length of the $j^{th}$ structural member is denoted by $L_{j}$. The term ρ corresponds to the material’s or weight density. The experimental results are presented in Table 7. The average optimization result of IRBMO on this problem was 5.2424 × 10^2^, ranking first. Its standard deviation was 4.6514 × 10^−4^, the smallest among all compared algorithms, indicating that IRBMO demonstrated very stable performance on the 10-bar truss design problem. The results of the Wilcoxon test show that the performance of IRBMO was significantly better than all other algorithms except LSHADE_SPACMA, with no significant difference between the performance of IRBMO and LSHADE_SPACMA.

### 5.6. Topology Optimization (TO)

The focus of this problem is optimizing material distribution for a set of loads in a design space, subject to constraints on system performance. The formulation relies on the power-law approach, which is mathematically stated as:

(26) $$\begin{array}{cc} \; & Minimize:\\ \; & f\left(\vec{x}\right)=U^{T}KU=\sum_{e=1}^{N}{\left(x_{e}\right)}^{p}u_{e}^{T}k_{0}u_{0}\\ \; & subject\;to:\\ \; & h_{1}\left(\vec{x}\right)=\frac{V(\vec{x})}{V_{0}}-f=0\\ \; & h_{2}\left(\vec{x}\right)=\mathrm{KU}-F=0\\ \; & with\;bounds:\\ \; & 0\leq x_{u}\leq 1,\;u\in 1,\;2,\;3,\;\ldots ,\;30 \end{array}$$

Within this formulation, the vectors *F* and *U* denote the force and global displacements, respectively. *K* corresponds to the global stiffness matrix, while $k_{e}$ and $u_{e}$ are the element-level stiffness matrix and displacement vector. The design variables are contained in the vector $\vec{x}$. Furthermore, *N* quantifies the total number of elements discretizing the design domain, and p(p=3) represents the penalization power. Finally, $V(\vec{x})$ indicates the material volume, $V_{0}$ is the total design domain volume, and *f* specifies the prescribed volume fraction.

The experimental results are shown in Table 8. The average result of IRBMO on this problem was 2.6393 × 10^0^, with a standard deviation of 1.2599 × 10^−12^. TO is a complex optimization problem with 30 constraints. IRBMO performed exceptionally well on this problem, achieving the best average result and a relatively small standard deviation. Additionally, the results of the Wilcoxon signed-rank test indicated that IRBMO’s performance was significantly superior to all other compared algorithms.

### 5.7. Summary of the COPs

In Section 5.3, Section 5.4, Section 5.5 and Section 5.6, the performance of 11 algorithms, including IRBMO, is evaluated on four COPs. The results include metrics such as the mean value, the best value, the mean rank, and the parameters corresponding to the best value. The experimental results show that, across the four given COPs, IRBMO consistently achieved the best average optimization results, with relatively small standard deviations, demonstrating exceptional convergence accuracy and stability. Additionally, the results of the Wilcoxon test indicate that IRBMO outperforms the compared algorithms in most cases. These findings suggest that IRBMO not only excels on benchmark functions but also possesses effective global search capabilities in constrained problem solution spaces, highlighting its potential for application in real-world engineering problems.

## 6. 3D Trajectory Planning for UAVs

Unmanned aerial vehicles (UAVs) are integral to a wide range of civilian and military operations. Their operational value and significance are broadly recognized [103]. Path planning serves as one of the core tasks in the autonomous control systems of UAVs, aiming to determine a reliable and safe route from the starting point to the target point under specific constraints. This task represents a highly complex constrained optimization problem. Driven by the widespread adoption of UAVs, their path planning has become a focal point of research. We apply the IRBMO method to optimize UAV trajectories. The algorithm’s effectiveness is validated, and the problem’s mathematical definition is detailed below:

### 6.1. IRBMO Is Used to UAV 3D Path Planning Modeling

High mountain peaks, adverse weather conditions, and restricted airspace impose the primary constraints on Unmanned Aerial Vehicle (UAV) flight paths within mountainous terrain. Generally, to ensure flight safety, UAVs need to navigate while avoiding these areas. Regarding the issue of UAV path planning in mountainous terrains, this paper comprehensively takes into account various factors including mountain peaks, meteorological threats, and no-fly zones, and constructs a corresponding path-planning model. The mathematical model used to describe the terrain and obstacles can be represented by Equation (27).

(27) $$z=\sin\left(y+1\right)+\sin\left(x\right)+\cos\left(x^{2}+y^{2}\right)+2\times \cos\left(y\right)+\sin\left(x^{2}+y^{2}\right)$$

UAV flight trajectories must adhere to specific constraints, principally Trajectory Length, Maximum Turning Angle, and Flight Altitude.

Trajectory Length: Since the primary objectives for UAV flights involve maximizing time efficiency and reducing costs (while ensuring safety), trajectory length emerges as a critical metric in path planning. Equation (28) presents the mathematical formulation for this objective.

(28) $$F_{pc}=\sum_{m=1}^{g-1}{\left\|\left(x_{m+1},y_{m+1},z_{m+1}\right)-\left(x_{m},y_{m},z_{m}\right)\right\|}_{2}$$

where $\left(x_{m},y_{m},z_{m}\right)$ corresponds to the coordinates of the $m^{th}$ waypoint on the UAV’s planned path.

Flight Altitude: The altitude at which a UAV operates significantly impacts both the control system and flight safety. The mathematical model representing this constraint is given in Equation (29).

(29) $$F_{hc}=\sqrt{\sum_{m=1}^{g}{\left(z_{m}-\frac{1}{n}\sum_{k=1}^{g}z_{m}\right)}^{2}}$$

Maximum Turning Angle: A constraint is imposed on the UAV’s turning angle to keep it within the maximum allowed value. This limitation is expressed as:

(30) $$F_{sc}=\sum_{m=1}^{g-2}\left(\arccos\left(\frac{\varphi_{m+1}\times \varphi_{m}}{|\varphi_{m+1}\left|\times \right|\varphi_{m}|}\right)\right)$$

Here, $\varphi_{m}$ represents $x_{m+1}-x_{m},y_{m+1}-y_{m},z_{m+1}-z_{m}$.

(31) $$F_{tc}=w_{1}\times F_{pc}+w_{2}\times F_{hc}+w_{3}\times F_{sc}$$

(32) $$\left\{\begin{array}{c} w_{i}\geq 0\\ \sum_{i=1}^{3}w_{i}=1 \end{array}\right.$$

The weights $w_{i}\left(i=1,2,3\right)$ are the weighting factors. Equation (32) defines the constraints on these weight coefficients. Adjusting these values modifies the influence of each factor on the trajectory.

From the expressions above, the complete mathematical model for the 3D UAV path planning problem, comprising the objective function and constraints, is derived and presented in Equation (33).

(33) $$\begin{array}{cc} \; & \min_{L}F_{tc}\left(L\right)\\ \; & s.t.path(L)\;\notin \;Ground\cup Obstacle \end{array}$$

Here, *L* denotes the path generated via cubic spline interpolation, Ground represents the ground, and Obstacle represents the obstacles. The term Ground∪Obstacle signifies their union. The objective function in Equation (33) is given by Equation (31), while the constraints require the path *L* to avoid the ground and obstacles.

### 6.2. Example of 3D Path Planning for UAV

In this study, we compared IRBMO with 15 other algorithms. These include the original RBMO, the LSHADE_SPACMA algorithm which won awards in the CEC competition, the highly - cited original algorithms, and their recently - proposed improved versions such as MELGWO, PPSO, WOA, GJO, HHO, SSA, as well as other advanced algorithms including DBO, FTTA, GBO [16], SMA [104], GTO, MFO, and SCA [12].

The test environment is defined as a continuous 200×200 square space, which contains seven mountain obstacles. The center coordinates and heights of these obstacles are detailed in Table 9. For paths that collide with obstacles, we employ the same penalty function method described in the engineering problem section, with the specific formula given in Equation (21). The weighting factors $w_{1}$, $w_{2}$, and $w_{3}$ for $F_{pc}$, $F_{hc}$, and $F_{sc}$ were specified as 0.4, 0.4, and 0.2, respectively. The UAV’s trajectory initiates at (0, 0, 20) and terminates at (200, 200, 30). We generated a feasible and smooth path, first by employing cubic spline interpolation and then by benchmarking against competing algorithms. We retained the experimental parameters from previous chapters. For all comparison algorithms, their hyperparameters were adopted from the original publications.

**Figure 31:**
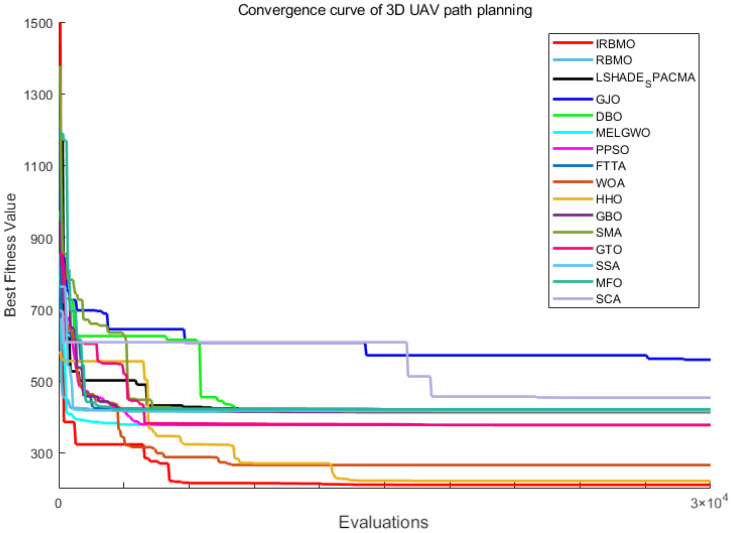
Optimal fitness search iteration curve.

**Figure 32:**
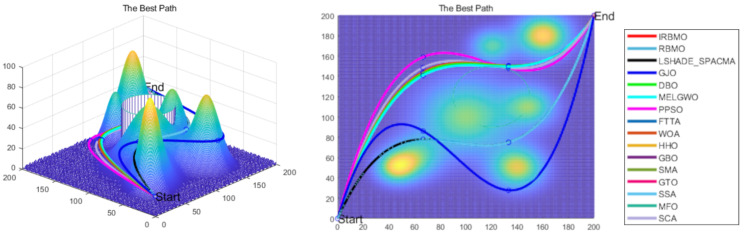
Flight tracks optimized by algorithms.

**Figure 33:**
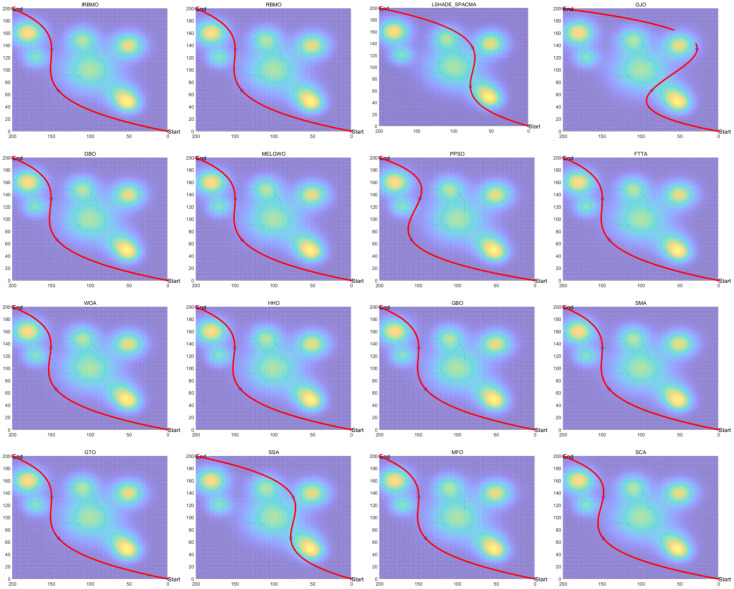
The generated UAV paths from fifteen algorithms.

The relevant experimental results are recorded in Table 10. Here, “Best” represents the $F_{tc}$ of the optimal path, “Mean” denotes the average path $F_{tc}$, “Worst” indicates the $F_{tc}$ of the worst-case path, “Std” stands for the standard deviation, and “Mean rank” represents the ranking based on the average path $F_{tc}$. Additionally, “Wilcoxon” denotes the *p*-value of the Wilcoxon test between IRBMO and the comparison algorithm; (+) indicates IRBMO is significantly superior, (−) indicates the comparison algorithm is significantly superior, and (=) signifies no significant difference. Figure 31 depicts the convergence curves of the 16 algorithms, while Figure 32 presents the 2D and 3D schematic diagrams of the optimal paths obtained through the 16 algorithms. To enhance the visual clarity of the paths, Figure 33 provides a comparative bird’s-eye view illustration of optimal path configurations generated by 16 distinct algorithms.

As shown in Table 10, the proposed IRBMO demonstrates superior performance in 3D UAV path planning. Three key observations emerge from the quantitative analysis:

- IRBMO, with a mean $F_{tc}$ of 352.540, significantly outperforms 14 competing algorithms, a finding supported by a Wilcoxon test with p<0.05. The only exception is GBO, with which no statistical difference was observed, as indicated by a *p*-value of 0.104. Notably, IRBMO’s best $F_{tc}$ value of 210.721 is identical to that of GBO. However, its worst $F_{tc}$ of 413.319 represents a 0.17% improvement over GBO’s 414.025, demonstrating superior adaptability in extreme scenarios.
- IRBMO’s standard deviation of 97.863 is 15.3% lower than RBMO’s value of 114.993, exhibiting the minimal fluctuation among the top-five algorithms based on mean rank. This stability is crucial for safety-critical UAV applications. This advantage is further highlighted when contrasted with WOA’s high standard deviation of 176.814, making IRBMO’s reliability more apparent.
- IRBMO’s worst-case result of 413.319 demonstrates a substantial improvement, outperforming GJO’s result of 556.564 by 25.7% and the traditional RBMO method’s 554.284 by 25.4%.

In addition, Figure 31 shows the convergence curves, from which it is evident that IRBMO has a faster convergence speed. Meanwhile, in Figure 32 and Figure 33, the trajectory obtained by IRBMO is the safest and relatively smooth, whereas the trajectory of GJO is the poorest, requiring multiple turn - backs to avoid threat areas. The experimental results demonstrate that the IRBMO algorithm boosts trajectory planning efficiency and exhibits distinct advantages.

## 7. Conclusions

This paper proposes an improved Red-billed Blue Magpie Optimizer (IRBMO) to address the limitations of the original RBMO algorithm, specifically its tendency to become trapped in local optima and insufficient global exploration capabilities when solving complex optimization problems.

First, a Logistic-tent chaotic map is introduced to initialize the population. Compared to the random initialization in the standard RBMO, this strategy significantly enhances the diversity of the initial population, enabling the algorithm to more uniformly explore the entire solution space during the initial search phase, thereby laying a solid foundation for subsequent global optimization and rapid convergence.

Second, to overcome convergence stagnation caused by the original algorithm’s over reliance on population mean values, a dynamic balance factor is introduced during the search phase. This factor dynamically adjusts the weights of the global optimal solution and population mean in guiding search directions, thereby achieving a better equilibrium between exploration and exploitation. Furthermore, a hybrid strategy integrating the Jacobi Curve and Lévy Flight is proposed. The long- and short-step jumping characteristics of Lévy Flight enhance the algorithm’s global perturbation capability, while the Jacobi Curve provides a nonlinear local perturbation mechanism. Combined, these mechanisms significantly improve the algorithm’s ability to escape local optima traps during late-stage convergence.

To comprehensively validate the performance of IRBMO, comprehensive comparisons were conducted against 15 benchmark algorithms, including classical RBMO, CEC competition-winning algorithms, and state-of-the-art metaheuristics proposed in recent years, using the CEC-2017 (30-, 50-, and 100-dimensional) and CEC-2022 (10- and 20-dimensional) test suites. Experimental results demonstrate that IRBMO achieves superior performance on most test functions, with significantly enhanced convergence accuracy, stability, and robustness compared to competitors, particularly excelling in high-dimensional complex problems. Furthermore, IRBMO was applied to four classical constrained engineering design problems and a complex 3D UAV path planning task. Results confirm its potential for practical engineering applications, as it consistently identifies higher-quality solutions.

While IRBMO exhibits exceptional performance in this study, several limitations warrant future exploration. For instance, its convergence speed on specific multimodal functions shows marginal room for improvement compared to optimal benchmarks. Future work will focus on developing parameter self-adaptation mechanisms to strengthen algorithmic universality and extend IRBMO’s applicability to broader domains, such as multi-objective optimization, neural architecture search, and large-scale scheduling problems.

## Figures and Tables

**Table 1:** The parameter settings for compared algorithms.

Algorithms	Parameter	Value	Algorithms	Parameter	Value
RBMO	—	—	AVOA	$L_{1}$	[0.7, 0.9]
DE	F; CR	0.8; 0.1		$L_{2}$	[0.1, 0.3]
LSHADE	Pb; Arc_rate	0.1; 2		*w*	[2, 3]
LSHADE_SPACMA	Pb	0.1		$P_{1}$	[0.4, 0.6]
	Arc_rate	2		$P_{2}$	[0.4, 0.6]
	L_rate	0.8		$P_{3}$	[0.4, 0.6]
LSHADE-cnEpSin	pb; ps	0.4; 0.5	SO	$c_{1}$; $c_{2}$; $c_{3}$	0.5; 0.05; 0.2
	structure	{2, 4, 10, 14}		Threshold	0.25
PPSO	*p*	0.02		Threshold2	0.6
	Crossover	0.6	GTO	p;β;w	0.03; 3; 8
MELGWO	*a*	[0, 2]	BKA	P; r	0.9; [0, 1]
	Crossover	0.6	HO	—	—
	SL_Search	0.5	RIME	*W*	5
WOA	*a*	Linearly2to0	FTTA	tp	tdistribution
	*b*	1	HHO	$E_{0}$	(−1,1)
SSA	$c_{1}$	Linearly2to0	GBO	$pr;\;\beta_{min};\;\beta_{max}$	0.5; 0.2; 1.2
GJO	$c_{1}$	1.5	MFO	*a*	Linearly−1to−2
DBO	k; b; s	0.1; 0.3; 0.5	SCA	*a*	2

**Table 2:** Description of the CEC-2017 test set.

Type	No.	CEC-2017 Function Name	Range	Dimension	$f_{\min}$
Unimodal	F1	Shifted and rotated bent cigar function	[−100, 100]	30/50/100	100
	F3	Shifted and rotated Zakharov function	[−100, 100]	30/50/100	300
Multimodal	F4	Shifted and rotated Rosenbrock’s function	[−100, 100]	30/50/100	400
	F5	Shifted and rotated Rastrigin’s Function	[−100, 100]	30/50/100	500
	F6	Shifted and rotated expanded Scaffer’s F6 Function	[−100, 100]	30/50/100	600
	F7	Shifted and ROTATED Lunacek Bi-Rastrigin function	[−100, 100]	30/50/100	700
	F8	Shifted and rotated non-continuous Rastrigin’s function	[−100, 100]	30/50/100	800
	F9	Shifted and rotated lévy function	[−100, 100]	30/50/100	900
	F10	Shifted and rotated Schwefel’s function	[−100, 100]	30/50/100	1000
Hybrid	F11	Hybrid function 1 (N = 3)	[−100, 100]	30/50/100	1100
	F12	Hybrid function 2 (N = 3)	[−100, 100]	30/50/100	1200
	F13	Hybrid function 3 (N = 3)	[−100, 100]	30/50/100	1300
	F14	Hybrid function 4 (N = 4)	[−100, 100]	30/50/100	1400
	F15	Hybrid function 5 (N = 4)	[−100, 100]	30/50/100	1500
	F16	Hybrid function 6 (N = 4)	[−100, 100]	30/50/100	1600
	F17	Hybrid function 6 (N = 5)	[−100, 100]	30/50/100	1700
	F18	Hybrid function 6 (N = 5)	[−100, 100]	30/50/100	1800
	F19	Hybrid function 6 (N = 5)	[−100, 100]	30/50/100	1900
	F20	Hybrid function 6 (N = 6)	[−100, 100]	30/50/100	2000
Composition	F21	Composition function 1 (N = 3)	[−100, 100]	30/50/100	2100
	F22	Composition function 2 (N = 3)	[−100, 100]	30/50/100	2200
	F23	Composition function 3 (N = 4)	[−100, 100]	30/50/100	2300
	F24	Composition function 4 (N = 4)	[−100, 100]	30/50/100	2400
	F25	Composition function 5 (N = 5)	[−100, 100]	30/50/100	2500
	F26	Composition function 6 (N = 5)	[−100, 100]	30/50/100	2600
	F27	Composition function 7 (N = 6)	[−100, 100]	30/50/100	2700
	F28	Composition function 8 (N = 6)	[−100, 100]	30/50/100	2800
	F29	Composition function 9 (N = 3)	[−100, 100]	30/50/100	2900
	F30	Composition function 10 (N = 3)	[−100, 100]	30/50/100	3000

**Table 3:** Description of the CEC-2022 test set.

Type	No.	CEC-2022 Function Name	Range	Dimension	$f_{\min}$
Unimodal	F1	Shifted and full rotated Zakharov function	[−100, 100]	10/20	300
Multimodal	F2	Shifted and full rotated Rosenbrock’s function	[−100, 100]	10/20	400
	F3	Shifted and full rotated Rastrigin’s function	[−100, 100]	10/20	600
	F4	Shifted and full rotated non-continuous Rastrigin’s function	[−100, 100]	10/20	800
	F5	Shifted and full rotated lévy function	[−100, 100]	10/20	900
Hybrid	F6	Hybrid function 1 (N = 3)	[−100, 100]	10/20	1800
	F7	Hybrid function 2 (N = 6)	[−100, 100]	10/20	2000
	F8	Hybrid function 3 (N = 5)	[−100, 100]	10/20	2200
Composition	F9	Composition function 1 (N = 5)	[−100, 100]	10/20	2300
	F10	Composition function 2 (N = 4)	[−100, 100]	10/20	2400
	F11	Composition function 3 (N = 5)	[−100, 100]	10/20	2600
	F12	Composition function 4 (N = 6)	[−100, 100]	10/20	2700

**Table 4:** Details of the four real-world COPs.

No.	Name	*D*	$N_{g}$	$N_{h}$
1	Tension/Compression spring design (TCPD (case1))	3	4	0
2	Step-cone pulley problem (SCP)	5	8	3
3	10-bar truss design (10-BT)	10	3	0
4	Topology optimization (TO)	30	30	0

**Table 5:** The experimental results of Tension/Compression spring design.

Algorithm	Worst	Best	Std	Mean	Mean Rank	Wilcoxon
IRBMO	1.266 × 10^−2^	1.266 × 10^−2^	1.478 × 10^−7^	1.266 × 10^−2^	1	—
RBMO	1.266 × 10^−2^	1.266 × 10^−2^	4.837 × 10^−7^	1.266 × 10^−2^	2	0.025 (+)
LSHADE_SPACMA	1.280 × 10^−2^	1.266 × 10^−2^	4.715 × 10^−5^	1.269 × 10^−2^	6	0.007 (+)
LSHADE-cnEpSin	1.269 × 10^−2^	1.266 × 10^−2^	1.064 × 10^−5^	1.267 × 10^−2^	4	0.064 (=)
LSHADE	1.267 × 10^−2^	1.266 × 10^−2^	4.876 × 10^−6^	1.266 × 10^−2^	3	0.344 (=)
DE	1.280 × 10^−2^	1.266 × 10^−2^	4.484 × 10^−5^	1.268 × 10^−2^	5	0.140 (=)
HO	1.473 × 10^−2^	1.271 × 10^−2^	6.235 × 10^−4^	1.301 × 10^−2^	8	0.000 (+)
GJO	1.326 × 10^−2^	1.269 × 10^−2^	1.937 × 10^−4^	1.286 × 10^−2^	7	0.000 (+)
DBO	1.777 × 10^−2^	1.273 × 10^−2^	2.290 × 10^−3^	1.448 × 10^−2^	11	0.000 (+)
MELGWO	1.594 × 10^−2^	1.266 × 10^−2^	1.147 × 10^−3^	1.338 × 10^−2^	9	0.000 (+)
PPSO	1.777 × 10^−2^	1.267 × 10^−2^	1.981 × 10^−3^	1.404 × 10^−2^	10	0.000 (+)

**Table 6:** The experimental results of Step-cone pulley problem.

Algorithm	Worst	Best	Std	Mean	Mean Rank	Wilcoxon
IRBMO	1.607 × 10^1^	1.607 × 10^1^	1.369 × 10^−8^	1.607 × 10^1^	1	—
RBMO	1.607 × 10^1^	1.607 × 10^1^	1.163 × 10^−6^	1.607 × 10^1^	2	0.000 (+)
LSHADE_SPACMA	1.607 × 10^1^	1.607 × 10^1^	6.065 × 10^−9^	1.607 × 10^1^	4	0.021 (−)
LSHADE-cnEpSin	1.702 × 10^1^	1.612 × 10^1^	2.617 × 10^−1^	1.677 × 10^1^	8	0.000 (+)
LSHADE	1.607 × 10^1^	1.607 × 10^1^	7.804 × 10^−6^	1.607 × 10^1^	3	0.000 (+)
DE	7.052 × 10^93^	1.607 × 10^1^	2.230 × 10^93^	7.052 × 10^92^	5	0.000 (+)
HO	1.703 × 10^1^	1.649 × 10^1^	1.514 × 10^−1^	1.666 × 10^1^	7	0.000 (+)
GJO	3.100 × 10^94^	5.414 × 10^92^	9.821 × 10^93^	1.169 × 10^94^	10	0.000 (+)
DBO	9.855 × 10^95^	1.736 × 10^1^	3.740 × 10^95^	2.138 × 10^95^	11	0.000 (+)
MELGWO	1.708 × 10^1^	1.637 × 10^1^	2.266 × 10^−1^	1.667 × 10^1^	6	0.000 (+)
PPSO	1.711 × 10^1^	1.666 × 10^1^	1.751 × 10^−1^	1.702 × 10^1^	9	0.000 (+)

**Table 7:** The experimental results of 10-bar truss design.

Algorithm	Worst	Best	Std	Mean	Mean Rank	Wilcoxon
IRBMO	5.242 × 10^2^	5.242 × 10^2^	4.651 × 10^−4^	5.242 × 10^2^	1	—
RBMO	5.303 × 10^2^	5.242 × 10^2^	2.952 × 10^0^	5.260 × 10^2^	4	0.031 (+)
LSHADE_SPACMA	5.303 × 10^2^	5.242 × 10^2^	3.156 × 10^0^	5.266 × 10^2^	5	0.427 (=)
LSHADE_cnEpSin	5.303 × 10^2^	5.242 × 10^2^	2.576 × 10^0^	5.254 × 10^2^	3	0.000 (+)
LSHADE	5.243 × 10^2^	5.242 × 10^2^	3.938 × 10^−2^	5.242 × 10^2^	2	0.000 (+)
DE	5.304 × 10^2^	5.242 × 10^2^	2.942 × 10^0^	5.270 × 10^2^	6	0.000 (+)
HO	5.887 × 10^2^	5.285 × 10^2^	1.702 × 10^1^	5.433 × 10^2^	10	0.000 (+)
GJO	5.319 × 10^2^	5.252 × 10^2^	2.386 × 10^0^	5.274 × 10^2^	7	0.000 (+)
DBO	5.824 × 10^2^	5.246 × 10^2^	2.226 × 10^1^	5.478 × 10^2^	11	0.000 (+)
MELGWO	5.333 × 10^2^	5.253 × 10^2^	2.094 × 10^0^	5.309 × 10^2^	9	0.000 (+)
PPSO	5.351 × 10^2^	5.252 × 10^2^	3.744 × 10^0^	5.301 × 10^2^	8	0.000 (+)

**Table 8:** The experimental results of Topology optimization.

Algorithm	Worst	Best	Std	Mean	Mean Rank	Wilcoxon
IRBMO	2.639 × 10^0^	2.639 × 10^0^	1.259 × 10^−12^	2.639 × 10^0^	1	—
RBMO	2.639 × 10^0^	2.639 × 10^0^	2.943 × 10^−12^	2.639 × 10^0^	5	0.000 (+)
LSHADE_SPACMA	2.639 × 10^0^	2.639 × 10^0^	4.303 × 10^−12^	2.639 × 10^0^	6	0.000 (+)
LSHADE_cnEpSin	2.639 × 10^0^	2.639 × 10^0^	1.637 × 10^−8^	2.639 × 10^0^	7	0.000 (+)
LSHADE	2.645 × 10^0^	2.641 × 10^0^	1.556 × 10^−3^	2.643 × 10^0^	10	0.000 (+)
DE	2.641 × 10^0^	2.640 × 10^0^	6.273 × 10^−4^	2.640 × 10^0^	9	0.000 (+)
HO	2.639 × 10^0^	2.639 × 10^0^	4.681 × 10^−16^	2.639 × 10^0^	2	0.000 (+)
GJO	2.731 × 10^0^	2.681 × 10^0^	1.558 × 10^−2^	2.700 × 10^0^	11	0.000 (+)
DBO	2.639 × 10^0^	2.639 × 10^0^	4.681 × 10^−16^	2.639 × 10^0^	2	0.000 (+)
MELGWO	2.639 × 10^0^	2.639 × 10^0^	4.681 × 10^−16^	2.639 × 10^0^	2	0.000 (+)
PPSO	2.640 × 10^0^	2.639 × 10^0^	4.708 × 10^−4^	2.639 × 10^0^	8	0.000 (+)

**Table 9:** Parameters of the seven mountain peak obstacles.

Peak	Center	Height
1	(60, 60)	50
2	(100, 100)	60
3	(180, 160)	80
4	(50, 140)	70
5	(50, 45)	65
6	(110, 150)	54
7	(170, 120)	50

**Table 10:** Experimental results of 3D trajectory planning for UAV.

Algorithm	Worst	Best	Std	Mean	Mean Rank	Wilcoxon
IRBMO	413.319	210.721	97.863	352.540	1	—
RBMO	554.284	210.722	114.993	363.016	3	0.031 (+)
LSHADE_SPACMA	508.552	377.102	39.627	429.941	15	0.017 (+)
GJO	556.564	414.180	68.799	480.521	16	0.000 (+)
DBO	421.223	211.349	65.950	395.367	7	0.005 (+)
MELGWO	554.746	210.729	115.763	364.556	5	0.025 (+)
PPSO	422.250	281.802	44.548	402.864	9	0.005 (+)
FTTA	420.942	210.721	87.220	376.096	6	0.021 (+)
WOA	631.301	211.958	176.814	412.785	11	0.031 (+)
HHO	577.509	211.408	107.832	413.982	12	0.021 (+)
GBO	414.025	210.721	97.996	352.732	2	0.104 (=)
SMA	554.328	210.730	156.862	423.549	13	0.031 (+)
GTO	640.862	210.721	121.023	398.390	8	0.014 (+)
SSA	554.390	377.142	52.853	427.612	14	0.004 (+)
MFO	510.651	210.721	110.241	364.385	4	0.031 (+)
SCA	461.006	298.315	57.179	410.646	10	0.007 (+)

## Data Availability

All relevant data are within the paper.

## Supplementary Materials

The following supporting information can be downloaded at: https://www.mdpi.com/article/10.3390/biomimetics10110788/s1, Supplementary S1: The experimental results of the CEC test set. Table S1 presents the experimental results of 16 algorithms on the CEC benchmark sets, including CEC-2017 with 30, 50, 100 dimensions and CEC-2022 with 10, 20 dimensions. The names of the benchmark sets and their corresponding dimensions are indicated in the names of the worksheets at the bottom of the file. To visually highlight the algorithms with the best performance, the minimum value in each row is shown in bold. Supplementary S2: The results of Wilcoxon rank sum test for the CEC test set. Table S2 presents the results of the Wilcoxon rank-sum test between IRBMO and the other 15 comparison algorithms, based on the experimental data in Table S1. The names of the benchmark sets and their corresponding dimensions are likewise indicated in the worksheet names at the bottom of the file. Supplementary S3: Strategies Effectiveness and Parameter Sensitivity Analysis. Table S3 comprises three sheets, detailing: the ablation study results, the corresponding Wilcoxon test results, and the parameter sensitivity analysis results.
